# Nivolumab plus chemotherapy or ipilimumab in gastro-oesophageal cancer

**DOI:** 10.1038/s41586-022-04508-4

**Published:** 2022-03-23

**Authors:** Kohei Shitara, Jaffer A. Ajani, Markus Moehler, Marcelo Garrido, Carlos Gallardo, Lin Shen, Kensei Yamaguchi, Lucjan Wyrwicz, Tomasz Skoczylas, Arinilda Campos Bragagnoli, Tianshu Liu, Mustapha Tehfe, Elena Elimova, Ricardo Bruges, Thomas Zander, Sergio de Azevedo, Ruben Kowalyszyn, Roberto Pazo-Cid, Michael Schenker, James M. Cleary, Patricio Yanez, Kynan Feeney, Michalis V. Karamouzis, Valerie Poulart, Ming Lei, Hong Xiao, Kaoru Kondo, Mingshun Li, Yelena Y. Janjigian

**Affiliations:** 1 National Cancer Center Hospital East, Kashiwa, Japan; 2 The University of Texas MD Anderson Cancer Center, Houston, TX USA; 3 Johannes-Gutenberg University Clinic, Mainz, Germany; 4 Clinica San Carlos de Apoquindo, Pontificia Universidad Católica, Santiago, Chile; 5 Fundación Arturo López Pérez, Providencia, Chile; 6 Department of Gastrointestinal Oncology, Key Laboratory of Carcinogenesis and Translational Research (Ministry of Education/Beijing), Peking University Cancer Hospital and Institute, Beijing, China; 7 The Cancer Institute Hospital of JFCR, Tokyo, Japan; 8 Klinika Onkologii i Radioterapii, Narodowy Instytut Onkologii, Warszawa, Poland; 9 II Klinika Chirurgii Ogólnej, Gastroenterologicznej i Nowotworów Układu Pokarmowego, Medical University of Lublin, Lublin, Poland; 10 Fundacao Pio Xii Hospital Cancer De Barretos, Barretos, Brazil; 11 Zhongshan Hospital Fudan University, Shanghai, China; 12 Oncology Center – Centre Hospitalier de l’Universite de Montreal, Montreal, Canada; 13 Princess Margaret Cancer Centre, Toronto, Ontario Canada; 14 Instituto Nacional de Cancerologia E.S.E., Bogotá, Colombia; 15 University of Cologne, Department of Internal Medicine, Center for Integrated Oncology Aachen Bonn Cologne Düesseldorf; Gastrointestinal Cancer Group Cologne (GCGC), Cologne, Germany; 16 Hospital de Clínicas de Porto Alegre, Porto Alegre, Brazil; 17 Instituto Multidisciplinario de Oncologia, Clinica Viedma S.A., Viedma, Argentina; 18 Hospital Universitario Miguel Servet, Zaragoza, Spain; 19 SF Nectarie Oncology Center, Craiova, Romania; 20 Dana Farber Cancer Institute, Boston, MA USA; 21 Universidad de La Frontera, James Lind Cancer Research Center, Temuco, Chile; 22 St John of God Murdoch Hospital, Murdoch, Australia; 23 Laiko General Hospital of Athens, Athens, Greece; 24 Bristol Myers Squibb, Princeton, NJ USA; 25 Memorial Sloan Kettering Cancer Center and Weill Cornell Medical College, New York, NY USA

**Keywords:** Gastric cancer, Immunotherapy

## Abstract

Standard first-line chemotherapy results in disease progression and death within one year in most patients with human epidermal growth factor receptor 2 (HER2)-negative gastro-oesophageal adenocarcinoma^1–4^. Nivolumab plus chemotherapy demonstrated superior overall survival versus chemotherapy at 12-month follow-up in gastric, gastro-oesophageal junction or oesophageal adenocarcinoma in the randomized, global CheckMate 649 phase 3 trial^5^ (programmed death ligand-1 (PD-L1) combined positive score ≥5 and all randomized patients). On the basis of these results, nivolumab plus chemotherapy is now approved as a first-line treatment for these patients in many countries^6^. Nivolumab and the cytotoxic T-lymphocyte antigen-4 (CTLA-4) inhibitor ipilimumab have distinct but complementary mechanisms of action that contribute to the restoration of anti-tumour T-cell function and induction of de novo anti-tumour T-cell responses, respectively^7–11^. Treatment combining 1 mg kg^−1^ nivolumab with 3 mg kg^−1^ ipilimumab demonstrated clinically meaningful anti-tumour activity with a manageable safety profile in heavily pre-treated patients with advanced gastro-oesophageal cancer^12^. Here we report both long-term follow-up results comparing nivolumab plus chemotherapy versus chemotherapy alone and the first results comparing nivolumab plus ipilimumab versus chemotherapy alone from CheckMate 649. After the 24.0-month minimum follow-up, nivolumab plus chemotherapy continued to demonstrate improvement in overall survival versus chemotherapy alone in patients with PD-L1 combined positive score ≥5 (hazard ratio 0.70; 95% confidence interval 0.61, 0.81) and all randomized patients (hazard ratio 0.79; 95% confidence interval 0.71, 0.88). Overall survival in patients with PD-L1 combined positive score ≥ 5 for nivolumab plus ipilimumab versus chemotherapy alone did not meet the prespecified boundary for significance. No new safety signals were identified. Our results support the continued use of nivolumab plus chemotherapy as standard first-line treatment for advanced gastro-oesophageal adenocarcinoma.

## Abstract (web summary)

Results in the CheckMate 649 phase 3 trial for first-line combined nivolumab and chemotherapy treatment continue to show clinically meaningful efficacy in gastric, gastro-oesophageal junction or oesophageal adenocarcinoma after 24 months, with no new safety signals.

## Main

**Extended Data Fig. 1:**
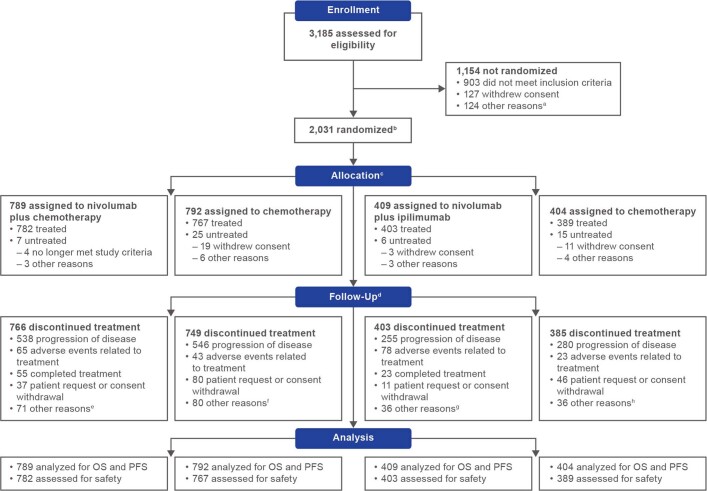
CONSORT diagram for patient disposition. ^a^Included death (*n* = 36), adverse events (*n* = 24), poor/noncompliance (*n* = 15), administrative reasons (*n* = 5), pregnancy (*n* = 1) and additional reasons (*n* = 43); ^b^Includes patients concurrently randomized to the nivolumab plus chemotherapy, nivolumab plus ipilimumab, and chemotherapy groups. Relevant protocol deviations were noted in 21 (1%) patients concurrently randomized to nivolumab plus chemotherapy versus chemotherapy: usage of prohibited on-treatment anti-cancer therapy (*n* = 12), baseline ECOG PS >1 (*n* = 5), incorrect cancer diagnosis (*n* = 2), prohibited prior anti-cancer therapy (at study entry) (*n* = 1) and no baseline (measurable or evaluable) disease (*n* = 1); Relevant protocol deviations were noted in 10 (1%) patients concurrently randomized to nivolumab plus ipilimumab versus chemotherapy: usage of prohibited on-treatment anti-cancer therapy (*n* = 5), incorrect cancer diagnosis (*n* = 2), no baseline PD-L1 result (*n* = 2) and baseline ECOG PS > 1 (*n* = 1); ^c^363 patients overlapped between the two chemotherapy groups from the 1:1:1 randomization period; ^d^The median follow-up for survival (time from concurrent randomization to last known date alive or death) was 13.1 months (range 0.1–49.5) and 11.2 months (range 0.0–47.9) in the nivolumab plus chemotherapy versus chemotherapy groups, respectively, and 11.4 months (range 0.0–52.1) and 11.5 months (range 0.0–52.8) in the nivolumab plus ipilimumab versus chemotherapy groups, respectively; ^e^Included adverse events unrelated to study treatment (*n* = 47), maximum clinical benefit (*n* = 11), lost to follow-up (*n* = 2), patient no longer met trial criteria (*n* = 1), poor/noncompliance (*n* = 1) and other reasons (*n* = 9); ^f^Included adverse events unrelated to study treatment (*n* = 35), maximum clinical benefit (*n* = 30), poor/noncompliance (*n* = 4), other reasons (*n* = 8), lost to follow-up (*n* = 2) and death (*n* = 1); ^g^Included adverse events unrelated to study treatment (*n* = 21), death (*n* = 3), poor/noncompliance (*n* = 1), other reasons (*n* = 6) and not reported (*n* = 5); ^h^Included adverse events unrelated to study treatment (*n* = 15), maximum clinical benefit (*n* = 13), lost to follow-up (*n* = 2), poor/noncompliance (*n* = 2) and other reasons (*n* = 4). ECOG PS, Eastern Cooperative Oncology Group performance status; OS, overall survival; PD-L1, programmed death ligand 1; PFS, progression-free survival.

We enrolled 3,185 patients, 2,031 of whom were randomized across the 3 treatment groups; of these, 1,581 patients were concurrently randomized to nivolumab plus chemotherapy or chemotherapy (April 2017 to May 2019) and 813 to nivolumab plus ipilimumab or chemotherapy (October 2016 to June 2018). Enrolment to the nivolumab-plus-ipilimumab group was closed early owing to increased rate of adverse events and early deaths relative to the other two study groups, per recommendation from the data monitoring committee. Among randomized patients, the number of patients who received one or more dose of study treatment and those that were discontinued at the data cut-off date for the current analysis (27 May 2021) are shown in Extended Data Fig. 1. The primary reason for treatment discontinuation was disease progression (Extended Data Fig. 1).

Baseline characteristics were balanced across the treatment groups (Supplementary Information). Most patients were of non-Asian race (≥70%) and had gastric cancer (≥69%), whereas approximately 18% and 12% had gastro-oesophageal junction (GEJ) cancer and oesophageal adenocarcinoma, respectively. Approximately 60% of patients across groups had tumours expressing PD-L1 combined positive score (CPS) ≥ 5 and 3% had microsatellite instability-high (MSI-H) tumours.

## Efficacy of nivolumab plus chemotherapy

**Fig. 1:**
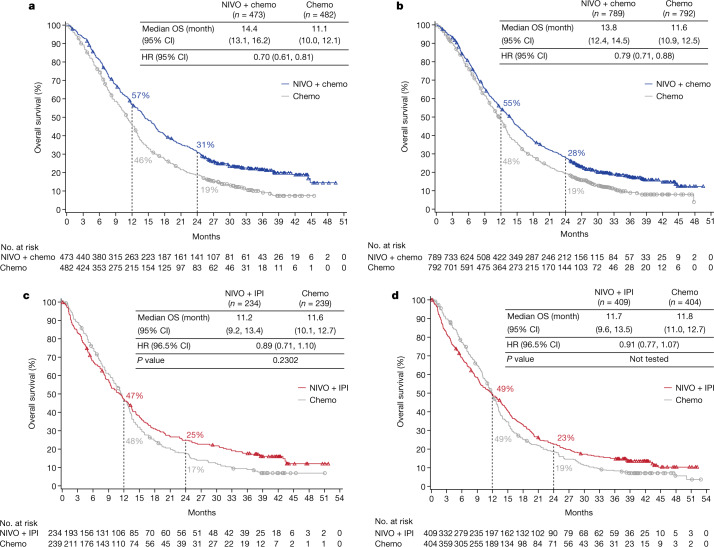
Kaplan–Meier estimates of overall survival. **a**, **b**, Overall survival with nivolumab plus chemotherapy versus chemotherapy in patients with PD-L1 CPS ≥ 5 (**a**) and in all randomized patients (**b**). Minimum follow-up, 24.0 months. **c**, **d**, Overall survival with nivolumab plus ipilimumab versus chemotherapy in patients with PD-L1 CPS ≥ 5 (**c**) and in all randomized patients (**d**). Minimum follow-up, 35.7 months. Chemo, chemotherapy; CI, confidence interval; HR, hazard ratio; IPI, ipilimumab; NIVO, nivolumab; OS, overall survival.

With a 24.0-month minimum follow-up (time from concurrent randomization of the last patient to clinical data cut-off), nivolumab plus chemotherapy continued to demonstrate improved overall survival versus chemotherapy in patients with PD-L1 CPS ≥ 5; median overall survival was 14.4 months (95% confidence interval 13.1, 16.2) versus 11.1 months (10.0, 12.1), respectively (Fig. 1a). There was a 30% reduction in the risk of death (hazard ratio 0.70 (95% confidence interval 0.61, 0.81)) and sustained separation of Kaplan–Meier curves; the proportion of patients alive at 24 months was 31% versus 19%, respectively. Similarly, improved overall survival with nivolumab plus chemotherapy versus chemotherapy was observed in all randomized patients; median overall survival was 13.8 months (95% confidence interval 12.4, 14.5) versus 11.6 months (95% confidence interval 10.9, 12.5), respectively, with a 21% reduction in the risk of death versus chemotherapy (hazard ratio 0.79; 95% confidence interval 0.71, 0.88) (Fig. 1b).

**Extended Data Fig. 2:**
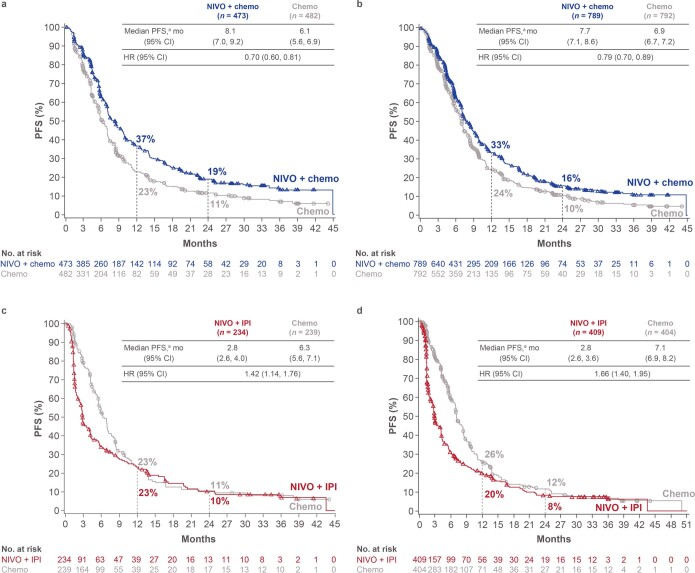
Kaplan-Meier estimates of progression-free survival. Progression-free survival with nivolumab plus chemotherapy versus chemotherapy in patients with PD-L1 CPS ≥ 5 (**a**) and in all randomized patients (**b**). Progression-free survival with nivolumab plus ipilimumab versus chemotherapy in patients with PD-L1 CPS ≥ 5 (**c**) and in all randomized patients (**d**). ^a^Per BICR. BICR, blinded independent central review; chemo, chemotherapy; CI, confidence interval; CPS, combined positive score; HR, hazard ratio; IPI, ipilimumab; mo, months; NIVO, nivolumab; PD-L1, programmed death ligand 1; PFS, progression-free survival.

**Extended Data Fig. 3:**
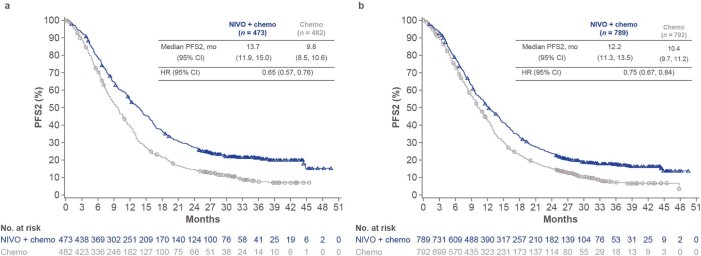
Kaplan-Meier plot of progression-free survival on subsequent therapy with nivolumab plus chemotherapy versus chemotherapy. **a**, Patients with PD-L1 CPS ≥ 5. **b**, All randomized patients. Chemo, chemotherapy; CI, confidence interval; CPS, combined positive score; HR, hazard ratio; mo, months; NIVO, nivolumab; PD-L1, programmed death ligand 1; PFS2, time from randomization to progression after subsequent systemic therapy, initiation of second subsequent systemic therapy, or death, whichever is earlier.

Progression-free survival (PFS) benefit was maintained after longer follow-up with nivolumab plus chemotherapy versus chemotherapy in patients with PD-L1 CPS ≥5 (hazard ratio 0.70; 95% confidence interval 0.60, 0.81) and in all randomized patients (hazard ratio 0.79; 95% confidence interval 0.70, 0.89); 24-month PFS rates were numerically higher in both populations (19% versus 11% and 16% versus 10%, respectively) (Extended Data Fig. 2a, b). Median PFS2 (time from randomization to progression after subsequent systemic therapy, initiation of second subsequent systemic therapy or death, whichever is earlier) was numerically longer with nivolumab plus chemotherapy versus chemotherapy (PD-L1 CPS ≥ 5, 13.7 months (95% confidence interval 11.9, 15.0) versus 9.8 months (8.5, 10.6; hazard ratio 0.65; 95% confidence interval 0.57, 0.76); all randomized patients, 12.2 months (95% confidence interval 11.3, 13.5) versus 10.4 months (9.7, 11.2; hazard ratio 0.75; 95% confidence interval 0.67, 0.84)) (Extended Data Fig. 3).

**Fig. 2:**
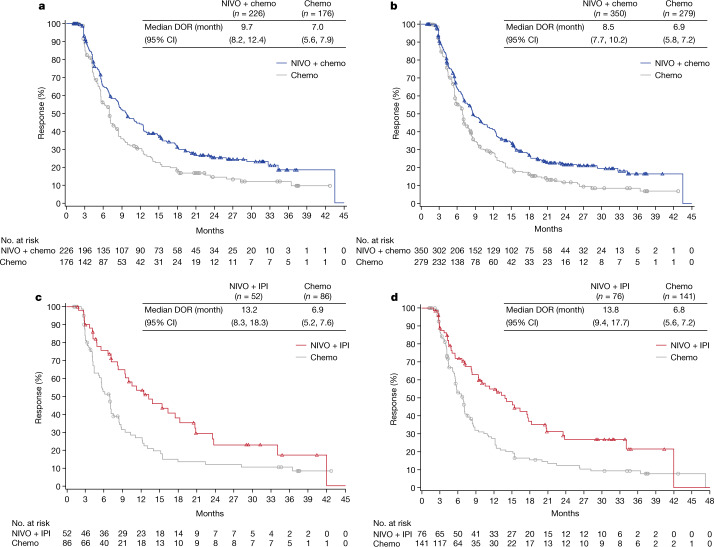
Kaplan–Meier estimates of duration of response. **a**, **b**, Duration of response per BICR with nivolumab plus chemotherapy versus chemotherapy in patients with PD-L1 CPS ≥ 5 (**a**) and in all randomized patients (**b**). **c**, **d**, Duration of response with nivolumab plus ipilimumab versus chemotherapy in patients with PD-L1 CPS ≥ 5 (**c**) and in all randomized patients (**d**). Number of responders (*n*) is indicated. Number of randomized patients who had target lesion measurements at baseline per BICR assessment for PD-L1 CPS ≥ 5: NIVO + chemo, *n* = 378; chemo, *n* = 390; all randomized: NIVO + chemo, *n* = 603; chemo, *n* = 607; PD-L1 CPS ≥ 5: NIVO + IPI, *n* = 196; chemo, *n* = 183; and all randomized: NIVO + IPI, *n* = 333; chemo, *n* = 299. BICR, blinded independent central review; DOR, duration of response.

**Extended Data Fig. 4:**
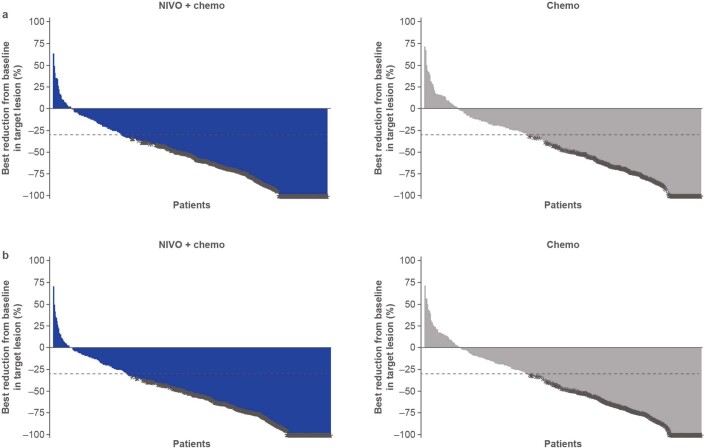
Waterfall plot of best percentage reduction in tumour burden with nivolumab plus chemotherapy versus chemotherapy. **a**, Patients with PD-L1 CPS ≥ 5. **b**, All randomized patients. Patients who had measurable disease at baseline per BICR and at least one on-treatment tumour assessment. Best reduction is maximum reduction in sum of diameters of target lesions. Horizontal reference line indicates the 30% reduction consistent with a RECIST v1.1 response. Asterisk symbol represents responders. Blue bars indicate nivolumab plus chemotherapy; grey bars indicate chemotherapy. BICR, blinded independent central review; chemo, chemotherapy; CPS, combined positive score; NIVO, nivolumab; PD-L1, programmed death ligand 1; RECIST, Response Evaluation Criteria in Solid Tumors.

Objective responses with nivolumab plus chemotherapy were observed in 226 (60%; 95% confidence interval 55, 65) of 378 patients with PD-L1 CPS ≥ 5 compared with 176 (45%; 95% confidence interval 40, 50) of 390 patients with chemotherapy. In all randomized patients, objective responses were observed in 350 (58%; 95% confidence interval 54, 62) of 603 patients with nivolumab plus chemotherapy versus 279 (46%; 95% confidence interval 42, 50) of 607 patients with chemotherapy (Extended Data Table 1). Additional complete responses were observed with nivolumab plus chemotherapy compared with the prespecified interim analysis at 12-month follow-up (PD-L1 CPS ≥ 5, *n* = 5 and all randomized, *n* = 6); there were no additional complete responses with chemotherapy alone. The total number of complete responses observed with nivolumab plus chemotherapy was 49 (13%) in patients with PD-L1 CPS ≥ 5 and 65 (11%) in all randomized patients; a total of 26 (7%) and 38 (6%) patients experienced complete responses with chemotherapy, respectively. Median duration of response with nivolumab plus chemotherapy versus chemotherapy was 9.7 versus 7.0 months in patients with PD-L1 CPS ≥ 5 and 8.5 versus 6.9 months in all randomized patients, respectively (Fig. 2a, b, Extended Data Table 1). The percentage of patients with PD-L1 CPS ≥ 5 who had more than 50% tumour burden reduction was 53% with nivolumab plus chemotherapy and 44% with chemotherapy; the percentage of patients with more than 80% reduction was 27% and 18%, respectively, with consistent results in all randomized patients (Extended Data Fig. 4).

## Efficacy of nivolumab plus ipilimumab

The hierarchically tested secondary endpoint of overall survival with nivolumab plus ipilimumab versus chemotherapy in patients with PD-L1 CPS ≥ 5 did not meet the prespecified boundary for significance at 35.7-month minimum follow-up; median overall survival was 11.2 (95% confidence interval 9.2, 13.4) versus 11.6 (95% confidence interval 10.1, 12.7) months, respectively (hazard ratio 0.89; 96.5% confidence interval 0.71, 1.10; *P* = 0.2302); 1-year overall survival rates were 47% (95% confidence interval 40, 53) and 48% (95% confidence interval 41, 54; Fig. 1c). The secondary endpoint of overall survival in all randomized patients with nivolumab plus ipilimumab versus chemotherapy was not statistically tested; median overall survival was 11.7 (95% confidence interval 9.6, 13.5) versus 11.8 (95% confidence interval 11.0, 12.7) months, respectively (hazard ratio 0.91; 96.5% confidence interval 0.77, 1.07); 1-year overall survival rates were 49% (95% confidence interval 44, 54) and 49% (95% confidence interval 44, 53; Fig. 1d). The 24-month overall survival rates with nivolumab plus ipilimumab versus chemotherapy were 25% versus 17% in patients with PD-L1 CPS ≥ 5 and 23% versus 19% in all randomized patients, respectively.

PFS and objective response rate (ORR) were not improved with nivolumab plus ipilimumab versus chemotherapy in patients with PD-L1 CPS ≥ 5 or in all randomized patients (Extended Data Fig. 2c, d, Extended Data Table 1). However, responses were more durable with nivolumab plus ipilimumab versus chemotherapy in both PD-L1 CPS ≥ 5 (median duration of response, 13.2 versus 6.9 months, respectively) and in all randomized patients (median duration of response, 13.8 versus 6.8 months; Fig. 2c, d, Extended Data Table 1).

## Subgroup analyses

**Extended Data Fig. 5:**
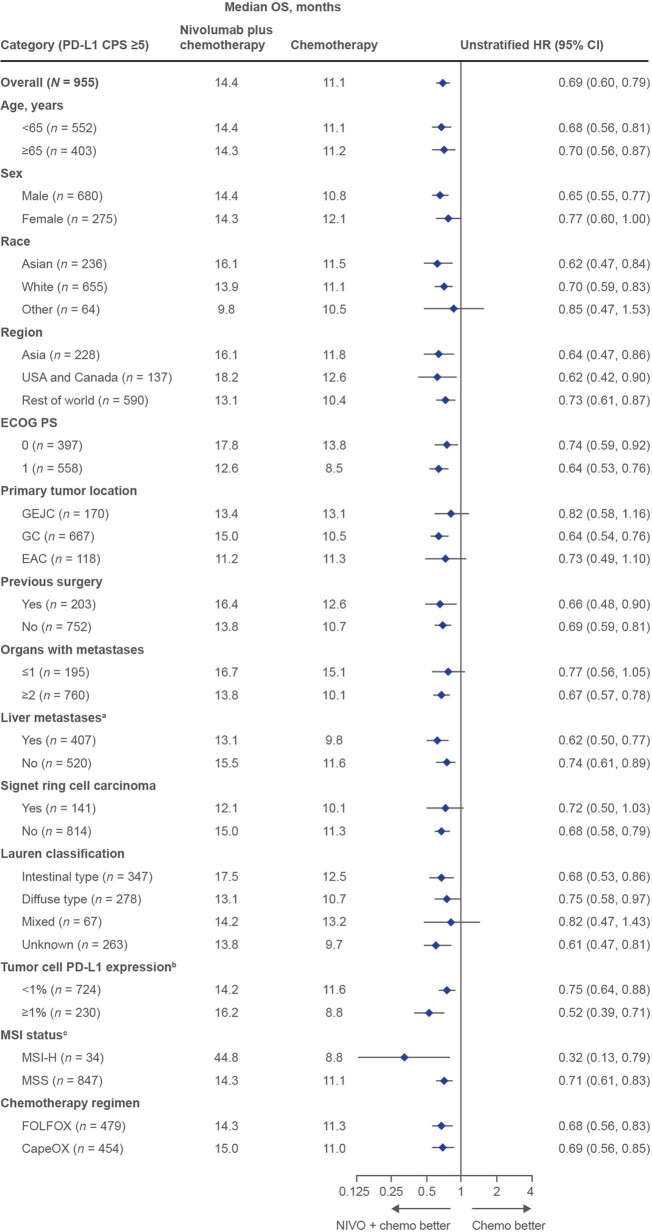
Forest plot of overall survival in prespecified subgroups with nivolumab plus chemotherapy versus chemotherapy in patients with PD-L1 CPS ≥ 5. ^a^Not reported, *n* = 28; ^b^Unknown, *n* = 1; ^c^Invalid/not reported, *n* = 74. Data are presented as unstratified hazard ratios and 95% CI. CapeOX, capecitabine plus oxaliplatin; chemo, chemotherapy; CI, confidence interval; CNS, central nervous system; CPS, combined positive score; EAC, oesophageal adenocarcinoma; ECOG PS, Eastern Cooperative Oncology Group performance status; FOLFOX, 5-fluorouracil plus leucovorin plus oxaliplatin; GC, gastric cancer; GEJC, gastro-oesophageal junction cancer; HR, hazard ratio; MSI, microsatellite instability; MSI-H, microsatellite instability-high; MSS, microsatellite stable; NIVO, nivolumab; OS, overall survival; PD-L1, programmed death ligand 1.

**Extended Data Fig. 6:**
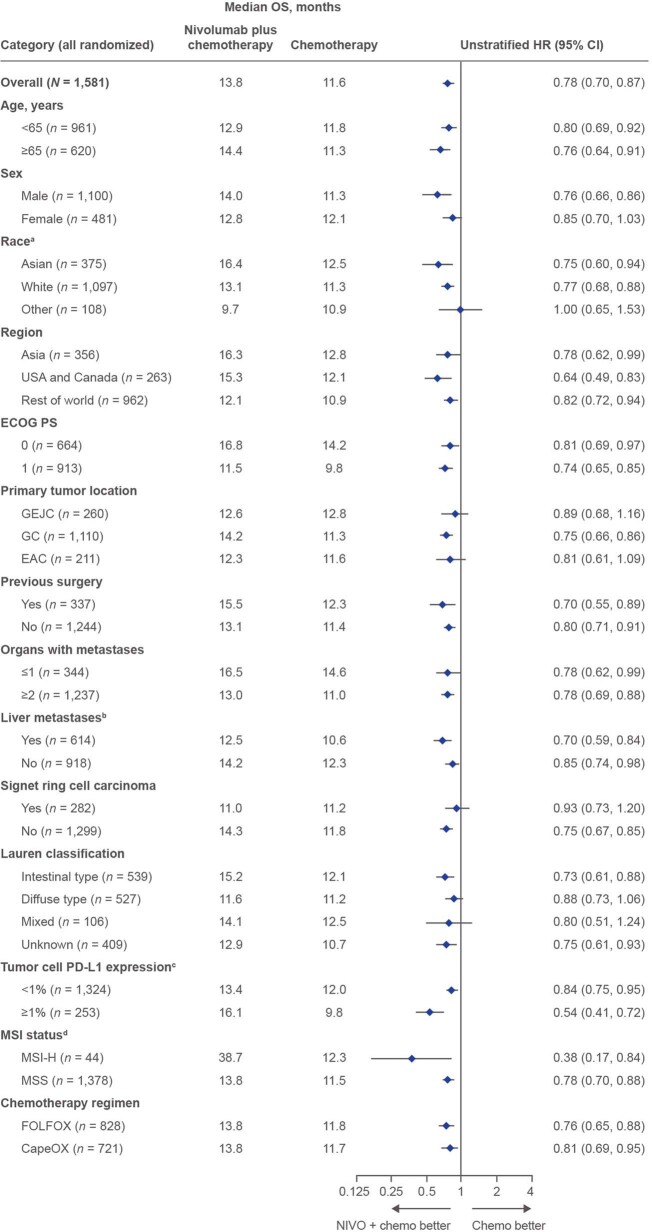
Forest plot of overall survival in prespecified subgroups with nivolumab plus chemotherapy versus chemotherapy in all randomized patients. ^a^Not reported, *n* = 1; ^b^Not reported, *n* = 49; ^c^Unknown, *n* = 4; ^d^Invalid/not reported, *n* = 159. Data are presented as unstratified HRs and 95% CI. CapeOX, capecitabine plus oxaliplatin; chemo, chemotherapy; CI, confidence interval; CNS, central nervous system; CPS, combined positive score; EAC, oesophageal adenocarcinoma; ECOG PS, Eastern Cooperative Oncology Group performance status; FOLFOX, 5-fluorouracil plus leucovorin plus oxaliplatin; GC, gastric cancer; GEJC, gastro-oesophageal junction cancer; HR, hazard ratio; MSI, microsatellite instability; MSI-H, microsatellite instability-high; MSS, microsatellite stable; NIVO, nivolumab; OS, overall survival; PD-L1, programmed death ligand 1.

**Extended Data Fig. 7:**
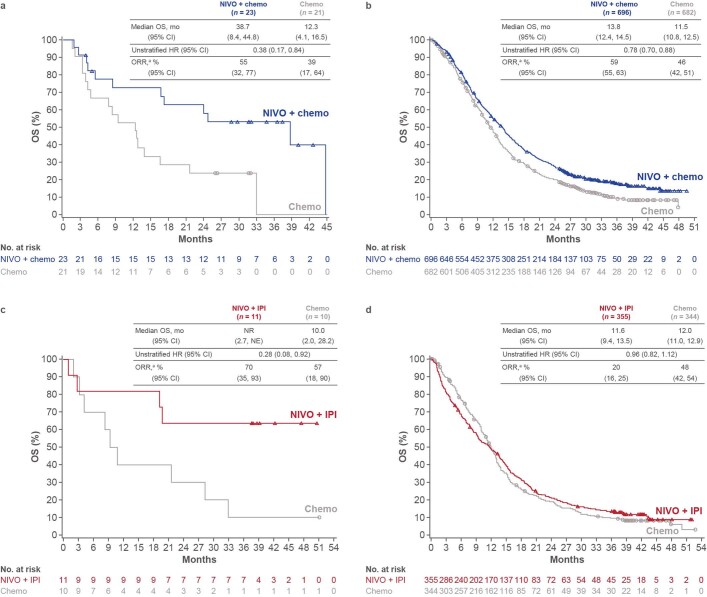
Kaplan-Meier estimates of overall survival and objective response rate by microsatellite instability status in all randomized patients. Overall survival with nivolumab plus chemotherapy in patients with MSI-H tumours (**a**) and those with MSS tumours (**b**). Overall survival with nivolumab plus ipilimumab in patients with MSI-H tumours (**c**) and those with MSS tumours (**d**). ^a^Randomized patients who had target lesion measurements at baseline per BICR assessment were **a**, NIVO + chemo, *n* = 20; Chemo, *n* = 18; **b**, NIVO + chemo, *n* = 535; Chemo, *n* = 533; **c**, Patients with MSI-H: NIVO + IPI, *n* = 10; Chemo, *n* = 7. **d**, NIVO + IPI, *n* = 292; Chemo, *n* = 257. Chemo, chemotherapy; CI, confidence interval; HR, hazard ratio; MSI-H, microsatellite instability-high; MSS, microsatellite stable; NIVO, nivolumab; ORR, objective response rate; OS, overall survival.

The hazard ratios for overall survival continued to favour nivolumab plus chemotherapy versus chemotherapy across multiple prespecified subgroups in patients with PD-L1 CPS ≥ 5 and all randomized patients with longer follow-up (Extended Data Figs. 5, 6). Overall survival benefit was enriched in patients with MSI-H tumours with nivolumab plus chemotherapy versus chemotherapy (unstratified hazard ratio 0.38; 95% confidence interval 0.17, 0.84; Extended Data Figs. 6, 7a); overall survival benefit in patients with microsatellite stable (MSS) tumours was consistent with that observed in all randomized patients (unstratified hazard ratio 0.78; 95% confidence interval 0.70, 0.88; Extended Data Figs. 6, 7b). ORR was also higher with nivolumab plus chemotherapy versus chemotherapy in patients with MSI-H tumours (55%; 95% confidence interval 32, 77 versus 39%; 95% confidence interval 17, 64, respectively) and those with MSS tumours (59%; 95% confidence interval 55, 63 versus 46%; 95% confidence interval 42, 51; Extended Data Fig. 7a, b). Similarly, nivolumab plus ipilimumab showed longer median overall survival (unstratified hazard ratio 0.28; 95% confidence interval 0.08, 0.92) and higher ORR (70%; 95% confidence interval 35, 93 versus 57%; 95% confidence interval 18, 90) compared with chemotherapy in patients with MSI-H tumours (Extended Data Fig. 7c, d).

**Fig. 3:**
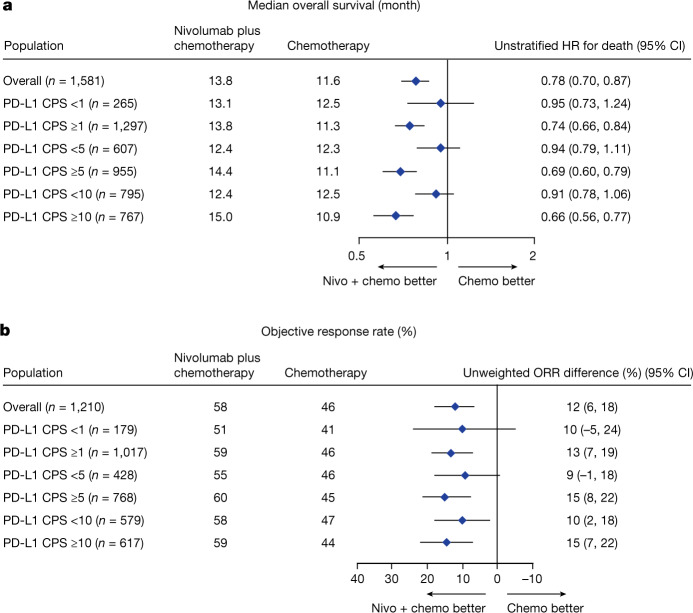
Forest plot of efficacy outcomes by PD-L1 CPS with nivolumab plus chemotherapy versus chemotherapy. **a**, Overall survival. PD-L1 CPS expression indeterminate, not evaluable or not reported for *n* = 19 patients. Data are presented as unstratified hazard ratios and 95% confidence interval. **b**, Objective response rate among randomized patients who had target lesion measurements at baseline, per blinded independent central review assessment. PD-L1 CPS expression indeterminate, not evaluable or not reported for *n* = 14 patients; percentages may not reflect an exact difference, owing to rounding. Data are presented as unweighted ORR differences and 95% confidence interval.

**Extended Data Fig. 8:**
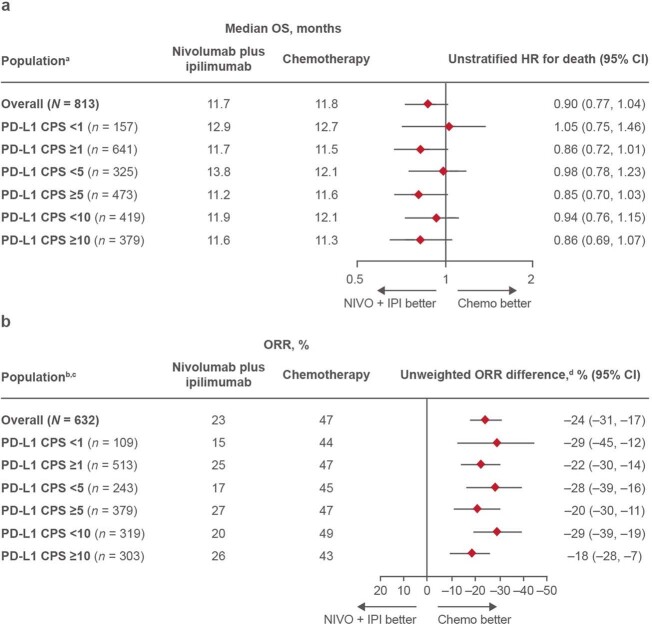
Forest plot of efficacy outcomes by PD-L1 CPS with nivolumab plus ipilimumab versus chemotherapy. **a**, Overall survival. Data are presented as unstratified HRs and 95% CI. **b**, Objective response rate. ^a^PD-L1 CPS expression indeterminate/not evaluable/not reported, *n* = 15; ^b^Randomized patients who had target lesion measurements at baseline, per blinded independent central review assessment; ^c^PD-L1 CPS expression indeterminate/not evaluable/not reported, *n* = 10; ^d^Percentages may not reflect an exact difference due to rounding. Data are presented as unweighted ORR differences  and 95% CI. Chemo, chemotherapy; CI, confidence interval; CPS, combined positive score; HR, hazard ratio; IPI, ipilimumab; NIVO, nivolumab; ORR, objective response rate; OS, overall survival; PD-L1, programmed death ligand 1.

The unstratified hazard ratios for overall survival with nivolumab plus chemotherapy in patients with PD-L1 CPS ≥ 10, ≥5 and ≥1 were 0.66 (95% confidence interval 0.56, 0.77), 0.69 (95% confidence interval 0.60, 0.79) and 0.74 (95% confidence interval 0.66, 0.84), respectively (Fig. 3a). In patients with PD-L1 CPS < 10, <5 and <1, the unstratified hazard ratios for overall survival were 0.91 (95% confidence interval 0.78, 1.06), 0.94 (95% confidence interval 0.79, 1.11) and 0.95 (95% confidence interval 0.73, 1.24), respectively (Fig. 3a). ORR was numerically higher with nivolumab plus chemotherapy versus chemotherapy across all evaluated PD-L1 CPS subgroups (Fig. 3b). Nivolumab plus ipilimumab did not show clear improvement in overall survival or ORR by PD-L1 CPS compared with chemotherapy (Extended Data Fig. 8).

## Subsequent therapy

Subsequent therapy was received by 41% and 44% of randomized patients in the nivolumab-plus-chemotherapy and chemotherapy groups and by 48% and 46% of patients in the nivolumab-plus-ipilimumab and chemotherapy groups. The most common subsequent treatment was chemotherapy (36% and 39% in the nivolumab-plus-chemotherapy and chemotherapy groups and 44% and 41% in the nivolumab-plus-ipilimumab and chemotherapy groups). Subsequent immunotherapy was received by 2% and 9% of patients in the nivolumab-plus-chemotherapy and chemotherapy groups and by 3% and 12% of patients in the nivolumab-plus-ipilimumab and chemotherapy groups (Supplementary Information).

## Safety

The median treatment duration was 6.8 months (range 0.1–45.0) and 4.9 months (range 0.0–44.2) with nivolumab plus chemotherapy versus chemotherapy and 1.9 months (range 0.0–24.1) and 4.9 months (range 0.1–45.5) with nivolumab plus ipilimumab versus chemotherapy (Supplementary Information). Grade 3–4 treatment-related adverse events (TRAEs) occurred in 60% and 45% of patients with nivolumab plus chemotherapy versus chemotherapy and in 38% and 46% with nivolumab plus ipilimumab versus chemotherapy (Table 1). The most common grade 3–4 TRAE was neutropaenia (15%) with nivolumab plus chemotherapy, increased lipase (7%) with nivolumab plus ipilimumab and neutropaenia (11–13%) with chemotherapy (Supplementary Information). TRAEs leading to discontinuation occurred in 38% and 25% of patients in the nivolumab-plus-chemotherapy versus chemotherapy groups, respectively, and in 22% and 26% of patients in the nivolumab-plus-ipilimumab versus chemotherapy groups (Table 1). Any-grade serious TRAEs were reported in 175 (22%) of 782 patients with nivolumab plus chemotherapy and 94 (12%) of 767 patients with chemotherapy and in 122 (30%) of 403 patients with nivolumab plus ipilimumab and 54 (14%) of 389 patients with chemotherapy. There were 16 treatment-related deaths with nivolumab plus chemotherapy, 10 with nivolumab plus ipilimumab and 5 with chemotherapy. The majority of TRAEs with a potential immunologic aetiology were grade 1 or 2; grade 3–4 events occurred in ≤5% of patients receiving nivolumab plus chemotherapy and in ≤12% of patients receiving nivolumab plus ipilimumab across organ categories (Supplementary Information).

**Table 1:** Summary of treatment-related adverse events in all treated patients

	Nivolumab plus chemotherapy (*n* = 782)^a,b^		Chemotherapy (*n* = 767)^a,b^		Nivolumab plus ipilimumab (*n* = 403)^a,c^		Chemotherapy (*n* = 389)^a,c^	
	Any grade	Grade 3–4	Any grade	Grade 3–4	Any grade	Grade 3–4	Any grade	Grade 3–4
All events	739 (95)	471 (60)	682 (89)	344 (45)	323 (80)	155 (38)	356 (92)	180 (46)
Serious events	175 (22)	133 (17)	94 (12)	77 (10)	122 (30)	93 (23)	54 (14)	45 (12)
Events leading to discontinuation^d^	300 (38)	141 (18)	188 (25)	70 (9)	88 (22)	68 (17)	101 (26)	37 (10)

Data are presented as *n* (%). There were 16 treatment-related deaths with nivolumab plus chemotherapy (4 events of pneumonitis, 2 events of febrile neutropaenia or neutropenic fever and 1 event each of acute cerebral infarction, disseminated intravascular coagulation, gastrointestinal bleeding, gastrointestinal toxicity, infection, intestinal mucositis, mesenteric thrombosis, pneumonia, septic shock and stroke), 10 with nivolumab plus ipilimumab (2 events of cardiac failure and 1 event each of acute hepatic failure, autoimmune hepatitis, general physical health deterioration, herpes simplex reactivation, hypophysitis, immune-mediated enterocolitis, multiple organ dysfunction syndrome, pneumonitis and upper gastrointestinal haemorrhage), and 5 with chemotherapy (1 event each of aesthenia and severe hyporexia, diarrhea, pancytopenia, pneumonitis and pulmonary thromboembolism). Treatment-related deaths were reported regardless of timeframe.

^a^Patients who received at least one dose of the assigned treatment. Includes events reported between first dose and 30 days after last dose of trial therapy. Treatment relatedness in the nivolumab-plus-chemotherapy group refers to nivolumab, at least one chemotherapy component or both and in the nivolumab-plus-ipilimumab group to nivolumab, ipilimumab or both. National Cancer Institute Common Terminology Criteria for Adverse Events, version 4.0, and Medical Dictionary for Regulatory Activities, version 23.0.

^b^Concurrently randomized to nivolumab plus chemotherapy versus chemotherapy.

^c^Concurrently randomized to nivolumab plus ipilimumab versus chemotherapy.

^d^Events leading to discontinuation of any drug in the regimen.

## Patient-reported outcomes

**Extended Data Fig. 9:**
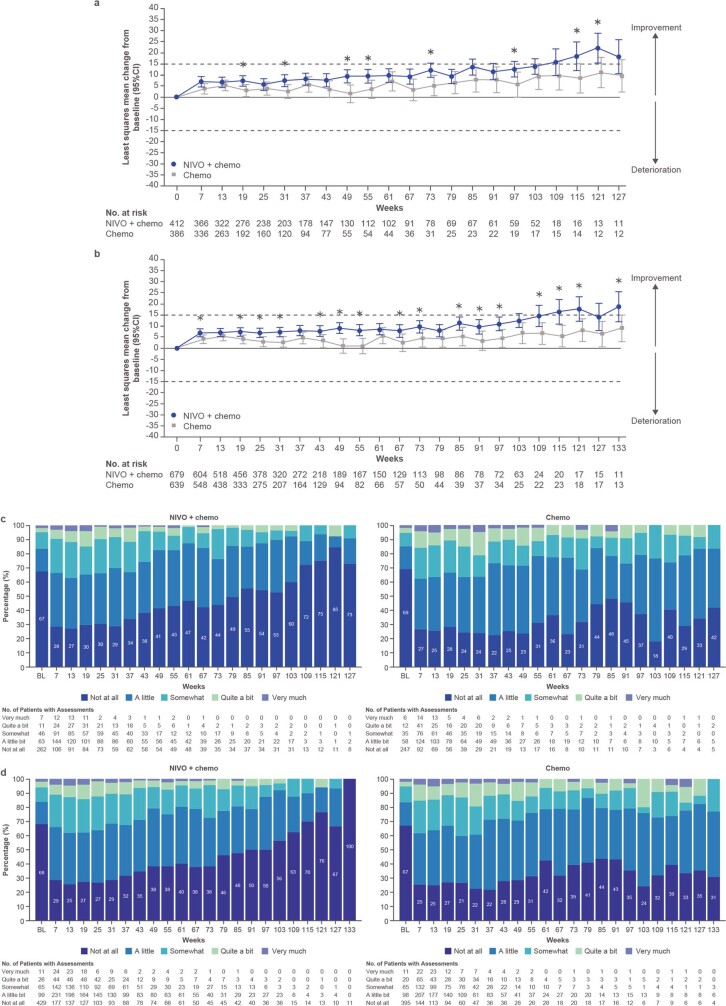
Patient-reported outcomes. Least squares mean (95% CI) change from baseline in FACT-Ga total score with nivolumab plus chemotherapy versus chemotherapy in patients with PD-L1 CPS ≥ 5 (nivolumab plus chemotherapy, *n* = 412; chemotherapy, *n* = 386) (**a**) and in all randomized patients (nivolumab plus chemotherapy, *n* = 679; chemotherapy, *n* = 639) (**b**). Data in panels **a** and **b** are presented as least squares mean change from baseline and 95% CI. Top and bottom dashed lines indicate minimally important difference in score. The primary meaningful change threshold is 15.1. The *P*-value for the difference in least squares means was computed as the two-tailed probability using the t distribution. No adjustments were made for multiple comparisons. *P < 0.05; in patients with PD-L1 CPS ≥ 5, *P*-value was 0.022 at week 19, 0.024 at week 31, 0.002 at week 49, 0.028 at week 55, 0.015 at week 73, 0.041 at week 97, 0.039 at week 115, and 0.025 at week 121. In all randomized patients, *P*-value was 0.026 at week 7, 0.020 at week 19, 0.012 at week 25, 0.006 at week 31, 0.025 at week 43, <0.001 at week 49, 0.002 at week 55, 0.037 at week 67, 0.030 at week 73, 0.033 at week 85, 0.028 at week 91, 0.012 at week 97, 0.024 at week 109, 0.004 at week 115, 0.013 at week 121, and 0.039 at week 133; not formally tested. FACT-Ga GP5 (“I am bothered by side effects of treatment”) item values in patients with PD-L1 CPS ≥ 5 (**c**) and in all randomized patients (**d**). Chemo, chemotherapy; CPS, combined positive score; FACT-Ga, Functional Assessment of Cancer Therapy-Gastric; NIVO, nivolumab; PD-L1, programmed death ligand 1.

Since the hierarchically tested secondary endpoint of overall survival with nivolumab plus ipilimumab versus chemotherapy in patients with PD-L1 CPS ≥ 5 was not met, the secondary endpoint of time to symptom deterioration (TTSD) in patients with PD-L1 CPS ≥ 5 and all randomized patients was not statistically tested. An improvement from baseline in the Functional Assessment of Cancer Therapy-Gastric (FACT-Ga) questionnaire total score was observed at all on-treatment assessments (Supplementary Information). The least-squares mean difference between treatment groups favoured nivolumab plus chemotherapy versus chemotherapy alone (at timepoints with ≥10 patients in each group); however, these differences did not reach the threshold for meaningful change (prespecified as 15.1 points; Extended Data Fig. 9a, b). The proportion of patients who reported not being bothered by treatment side effects over time on the basis of the GP5 item from FACT-Ga was higher with nivolumab plus chemotherapy than with chemotherapy alone, except at baseline when patients had not received treatment (Extended Data Fig. 9c, d).

## Discussion

Several targeted and immuno-oncology agents have been evaluated as first-line treatment for HER2-negative gastric or GEJ cancer; however, until recently, none have significantly prolonged survival relative to chemotherapy^1–4,13^. The positive results of CheckMate 649 reported with 12-month follow-up have established nivolumab plus chemotherapy as a standard first-line treatment for advanced gastric, GEJ or oesophageal adenocarcinoma^5^. After 24-month follow-up, nivolumab plus chemotherapy continued to demonstrate clinically meaningful improvement in overall survival, PFS and ORR versus chemotherapy alone. Hazard ratios for overall survival were directionally improved with nivolumab plus chemotherapy versus chemotherapy relative to the 12-month follow-up^5^ (PD-L1 CPS ≥ 5, 0.71; 98.4% confidence interval 0.59, 0.86; all randomized, 0.80; 99.3% confidence interval 0.68, 0.94), and 2-year survival rates were higher with nivolumab plus chemotherapy compared with chemotherapy. ORR was higher with nivolumab plus chemotherapy versus chemotherapy and a greater proportion of patients experienced reduction in tumour burden versus chemotherapy. Responses deepened with nivolumab plus chemotherapy with longer follow-up as evidenced by the additional complete responses compared with the 12-month follow-up.

There is considerable variation in the previously reported prevalence of PD-L1 CPS expression in gastro-oesophageal adenocarcinoma, with PD-L1 CPS ≥ 5 detected in 17–60% of patients^14–17^. CheckMate 649 is the most robust dataset to date to report PD-L1 CPS ≥ 5 prevalence using an analytically validated assay (28-8 pharmDx) in gastric, GEJ or oesophageal adenocarcinoma. The phase 3 ORIENT-16 trial in China reported a similar PD-L1 CPS ≥ 5 prevalence of approximately 60% using the 22C3 PharmDx assay^17,18^. In CheckMate 649, the magnitude of survival benefit continued to be enriched with nivolumab plus chemotherapy versus chemotherapy in patients with higher PD-L1 CPS, consistent with results at 12-month follow-up^5^. However, in patients with PD-L1 CPS ≥ 5 and ≥10, hazard ratios for overall survival were rather close to each other, with overlapping confidence intervals, and ORR benefit was similar, suggesting no meaningful further enrichment of clinical benefit at or above PD-L1 CPS 10. Furthermore, the ORRs observed with nivolumab plus chemotherapy were higher versus chemotherapy across all evaluated PD-L1 CPS cut-offs, suggesting that clinical benefit with nivolumab plus chemotherapy is not restricted to patients with PD-L1 CPS ≥ 5. Further analyses may help identify factors that are associated with higher magnitude of clinical benefit in patients with lower PD-L1 CPS.

Overall survival continued to favour nivolumab plus chemotherapy versus chemotherapy across multiple prespecified baseline characteristics with longer follow-up. Notably, the magnitude of survival benefit was markedly greater in the MSI-H subgroup for both patients with PD-L1 CPS ≥ 5 and all randomized patients, suggesting that benefit is independent of PD-L1 CPS. Similar results were reported with first-line pembrolizumab plus chemotherapy versus chemotherapy in patients with gastric or GEJ cancer (PD-L1 CPS ≥ 1) who had MSI-H tumours^13^. In CheckMate 649, the overall survival benefit in patients with MSS tumours was similar to that observed in all randomized patients.

The secondary endpoint of overall survival with nivolumab plus ipilimumab versus chemotherapy in CheckMate 649 did not meet the prespecified boundary for significance in patients with PD-L1 CPS ≥ 5. The observed response rates with nivolumab plus ipilimumab were lower versus chemotherapy, and there was no enrichment with increasing PD-L1 CPS cut-offs. However, the median duration of response almost doubled with nivolumab plus ipilimumab versus chemotherapy, which is consistent with results in other solid tumours with this combination^19–22^.

The lack of significant overall survival improvement with nivolumab plus ipilimumab is probably a result of multiple factors. There was an increase in early death rate with nivolumab plus ipilimumab versus chemotherapy; crossing of the Kaplan–Meier curves, which is a known phenomenon with immuno-oncology therapies^13,23,24^, was observed at 12 months, and the overall survival curves remained separated thereafter in favour of nivolumab plus ipilimumab. A higher number of patients receiving subsequent immuno-oncology therapy in the chemotherapy versus nivolumab-plus-ipilimumab group (12% versus 3%, respectively) may have also contributed to these results.

Tumours in gastric, GEJ or oesophageal adenocarcinoma are composed of distinct molecular subtypes^25,26^. Although dual checkpoint inhibition has been proven to be effective in multiple solid tumours^19–22,27^, further research is needed to evaluate how tumour biology, molecular heterogeneity, dynamics in tumour microenvironment and other patient factors may affect the efficacy of combined PD-L1 and CTLA-4 blockade. Notably, in the small but relevant subgroup of patients with microsatellite instability, which is characterized by high tumour mutational burden and CD8-positive T-cell infiltrates and is susceptible to immune-checkpoint inhibition^25,28–30^, longer overall survival and higher ORR were observed with nivolumab plus ipilimumab versus chemotherapy in CheckMate 649. These data suggest that combined immune checkpoint blockade in this patient population might be of interest to explore in future studies.

No new safety signals were identified with nivolumab plus chemotherapy in CheckMate 649 with similar frequencies of TRAEs relative to the 12-month follow-up. The safety profile of nivolumab plus ipilimumab observed in this trial was consistent with the known safety profile of this combination^12,27^. Limitations of this study have been previously discussed^5^.

In conclusion, the long-term clinically meaningful overall survival and PFS benefit, improved and durable responses, maintained health-related quality of life, and acceptable safety profile indicate a favourable benefit–risk profile of nivolumab plus chemotherapy. These results further support the use of this regimen as a standard first-line treatment in previously untreated patients with advanced gastric, GEJ or oesophageal adenocarcinoma.

## Methods

### Patients

Adults with unresectable advanced or metastatic gastric, GEJ or oesophageal adenocarcinoma were enrolled, regardless of PD-L1 expression. Patients with known HER2-positive status were excluded, and prior systemic therapy for metastatic disease was not allowed. Other key inclusion criteria were an Eastern Cooperative Oncology Group performance status score of 0 or 1 and the ability to provide a fresh or archival tumour sample to determine PD-L1 status. Additional details on study criteria have been previously described^5^.

### Trial design and treatments

CheckMate 649 (NCT02872116) is a randomized, open-label, multicentre, global phase 3 trial of nivolumab plus chemotherapy or ipilimumab versus chemotherapy alone, conducted at 175 hospitals and cancer centres in 29 countries across Asia, Australia, Europe, North America, and South America. Detailed study design and methods for the nivolumab-plus-chemotherapy versus chemotherapy groups have been previously described^5^. In brief, patients were initially randomized 1:1 to nivolumab plus ipilimumab or to chemotherapy from October 2016 to March 2017. The nivolumab-plus-chemotherapy group was added later, and the randomization was switched to 1:1:1 in March 2017. Enrolment to the nivolumab-plus-ipilimumab group was closed early in June 2018, and after this time, the randomization was switched to a 1:1 ratio of nivolumab plus chemotherapy versus chemotherapy to May 2019. Patients already randomized to nivolumab plus ipilimumab could continue treatment per protocol, but the data remained blinded until the pre-planned final analysis. During enrolment, the population for primary endpoints was amended to patients whose tumours expressed PD-L1 CPS ≥ 5 for the nivolumab-plus-chemotherapy versus chemotherapy groups, although patients continued to be enrolled regardless of PD-L1 expression. Additional randomization procedures and stratification by tumour cell PD-L1 status (≥1% versus <1% including indeterminate), region (Asia versus United States and Canada versus rest of world), Eastern Cooperative Oncology Group performance status score (0 versus 1) and type of chemotherapy (CapeOX versus FOLFOX) have been described^5^.

Patients were administered nivolumab (360 mg every 3 weeks or 240 mg every 2 weeks) with investigator’s choice of chemotherapy (CapeOX (oxaliplatin 130 mg m^−2^ on day 1 and capecitabine 1,000 mg m^−2^ orally twice daily on days 1–14) every 3 weeks or FOLFOX (leucovorin 400 mg m^−2^ on day 1, fluorouracil 400 mg m^−2^ on day 1 and 1,200 mg m^−2^ on days 1–2, and oxaliplatin 85 mg m^−2^ on day 1) every 2 weeks); nivolumab (1 mg kg^−1^) with ipilimumab (3 mg kg^−1^) every 3 weeks for 4 cycles, followed by nivolumab (240 mg every 2 weeks); or chemotherapy alone. The dosing for nivolumab 1 mg kg^−1^ plus ipilimumab 3 mg kg^−1^ was selected based on results of the CheckMate 032 study, where this regimen provided numerically higher ORR and longer median overall survival compared with nivolumab monotherapy or nivolumab 3 mg kg^−1^ plus ipilimumab 1 mg kg^−1^, along with a manageable safety profile in heavily pre-treated patients with advanced gastro-oesophageal adenocarcinoma^12^. Treatment was permitted until documented disease progression, unacceptable toxicity, withdrawal of consent or trial end. Nivolumab or ipilimumab were given for a maximum of two years. Patients receiving nivolumab in combination with chemotherapy or ipilimumab were permitted to continue treatment beyond initial disease progression (per Response Evaluation Criteria in Solid Tumors (RECIST), version 1.1), based on the investigator’s judgement, until subsequent progression. Dose reductions were not permitted for nivolumab and ipilimumab; dose reductions for chemotherapy were permitted per local standards. Dose delays were allowed for both groups to manage treatment-related toxicity. Nivolumab, ipilimumab, CapeOX and FOLFOX were provided by the sponsor except in certain countries where CapeOX and FOLFOX were procured commercially if allowed by local regulations. Additional details on discontinuation criteria have been previously described^5^.

The trial was conducted according to Good Clinical Practice guidelines developed by the International Council for Harmonisation and in compliance with the trial protocol (Supplementary Appendix). The trial protocol was approved by the institutional review boards or independent ethics committees at each site (NCT02872116). All patients provided written informed consent prior to trial participation per Declaration of Helsinki principles.

### Endpoints and assessments

The dual primary endpoints were overall survival (time from randomization to death) and PFS (time from randomization to the date of the first documented tumour progression (by blinded independent central review (BICR) per RECIST, version 1.1) or death) in the nivolumab-plus-chemotherapy versus chemotherapy groups^5^ in patients with PD-L1 CPS ≥ 5. Secondary endpoints that were hierarchically tested if the primary endpoints were met were overall survival in patients with PD-L1 CPS ≥ 1 and in all randomized patients in the nivolumab-plus-chemotherapy versus chemotherapy group and overall survival and TTSD in patients with PD-L1 CPS ≥ 5 and in all randomized patients in the nivolumab-plus-ipilimumab versus chemotherapy group. Other key secondary endpoints that were not formally tested included BICR-assessed PFS and ORR evaluated at different PD-L1 CPS cut-offs and in all randomized patients. Key exploratory endpoints included BICR-assessed duration of response; landmark survival rates; PFS2 (time from randomization to progression after subsequent systemic therapy, initiation of second subsequent systemic therapy, or death, whichever is earlier); biomarkers potentially predictive of efficacy; health-related quality of life; and safety and tolerability.

Tumours were assessed using computed tomography or magnetic resonance imaging per RECIST, version 1.1, at baseline, every 6 weeks from the start of cycle 1 for 48 weeks and every 12 weeks thereafter, until disease progression per BICR assessment. Adverse events were assessed throughout the treatment period and during follow-up according to the National Cancer Institute Common Terminology Criteria for Adverse Events, version 4.0.

### PRO analyses

FACT-Ga analysis was done for patients with PD-L1 CPS ≥ 5 and all randomly assigned patients who had an assessment at baseline (day 1, assessment before administration of treatment on day of first dose) and at least one subsequent assessment while on treatment. The questionnaire completion rate, defined as the proportion of questionnaires actually received out of the expected number, was calculated and summarized at each assessment point using descriptive statistics. Mean score and mean change from baseline for the FACT-Ga scale were estimated using mixed model for repeated measures. The change from baseline was modelled as a linear function of treatment groups; trial assessment; baseline score; trial stratification factors; interaction terms between treatment group and trial assessment; interaction terms between baseline score and trial assessment; and any potential confounders. A clinically meaningful difference was defined as a 15.1 or greater change from baseline in FACT-Ga total score^31^. The *P*-value for the difference in least squares means was computed as the two-tailed probability using the t distribution. No adjustments were made for multiple comparisons. In addition, treatment burden was assessed by the individual GP5 item of the FACT-Ga. The GP5 item reads, “I am bothered by side effects of treatment.” Frequencies and percentages of the GP5 item question responses (‘not at all’, ‘a little bit’, ‘somewhat’, ‘quite a bit’ and ‘very much’) were tabulated at each assessment point with ten or more study subjects in each group.

### Statistical analyses

Patients concurrently randomized to the nivolumab-plus-chemotherapy versus chemotherapy groups and the nivolumab-plus-ipilimumab versus chemotherapy groups were included in the respective final overall survival analyses. For the comparison of nivolumab plus chemotherapy and chemotherapy, patients randomized to chemotherapy before the nivolumab-plus-chemotherapy arm was introduced were not included in the analysis. For the comparison of nivolumab plus ipilimumab and chemotherapy, patients randomized to chemotherapy after the closure of nivolumab-plus-ipilimumab arm were not included in the analysis.

For nivolumab plus ipilimumab versus chemotherapy, the analysis of overall survival was pre-planned at a minimum follow-up of approximately 36 months, which corresponded with the pre-planned final analysis of overall survival for nivolumab plus chemotherapy versus chemotherapy at a 24-month minimum follow-up. Since the dual primary endpoints for nivolumab-plus-chemotherapy versus chemotherapy groups were met^5^, the secondary endpoint of overall survival in the nivolumab-plus-ipilimumab versus chemotherapy groups was hierarchically tested in patients with PD-L1 CPS ≥ 5 followed by all randomized patients. If overall survival in the nivolumab-plus-ipilimumab versus chemotherapy groups met the criteria for statistical significance, the secondary endpoint of TTSD in the nivolumab-plus-ipilimumab versus chemotherapy groups was planned to be hierarchically tested in patients with PD-L1 CPS ≥ 5 followed by all randomized patients. In the interim analysis, two-sided alpha levels of 0.02 and 0.03 (type I error) were allocated to the dual primary endpoints of PFS and overall survival, respectively. The comparison of secondary endpoints of overall survival and TTSD for nivolumab plus ipilimumab versus chemotherapy inherited alpha independently from the two primary endpoints (fraction of *α* transmitted = 0.035) and was tested once after 36 months in patients with PD-L1 CPS ≥ 5 followed by all randomized patients.

The statistical power estimation for the comparison of primary endpoints for the nivolumab-plus-chemotherapy versus the chemotherapy groups has been described previously^5^. Sample size calculations of the primary endpoints were based on simulations in East software, version 6.4.1. (Cytel). The prevalence of patients with PD-L1 CPS ≥ 5 was assumed to be 35% of all randomized patients, based on limited available data^14,24,32^, with 285 patients estimated in the nivolumab-plus-ipilimumab versus chemotherapy analysis. Based on new information from the CheckMate 649 trial, this PD-L1 CPS ≥ 5 prevalence was revised to 60% of all randomized patients, with 489 patients estimated in the nivolumab-plus-ipilimumab versus chemotherapy analysis. For overall survival, the hazard ratio was modelled as a four-piece hazard ratio with an average of 0.7. With 36-month minimum follow-up, it was expected that the 411 events would provide 93% power.

Median PFS, overall survival, and duration of response were estimated using Kaplan–Meier methods, and the corresponding two-sided 95% confidence intervals were calculated using the log–log transformation method. The stratified Cox proportional hazards regression model, with the randomization factors as the stratification factors and treatment group as a single covariate, was used to assess differences between treatment groups in overall survival and PFS. An O’Brien and Fleming *α*-spending function was employed to determine the hazard ratio for overall survival, using a stratified Cox proportional hazards model. Stratification factors recorded in an interactive web response system were used in the analysis.

The proportion of patients with an objective response and corresponding two-sided 95% confidence intervals were calculated using the Clopper-Pearson method.

Statistical analyses were performed using SAS software, version 9.4 (SAS Institute, Cary, NC).

### Reporting summary

Further information on research design is available in the Nature Research Reporting Summary linked to this paper.

## Online content

Any methods, additional references, Nature Research reporting summaries, source data, extended data, supplementary information, acknowledgements, peer review information; details of author contributions and competing interests; and statements of data and code availability are available at 10.1038/s41586-022-04508-4.

### Supplementary information

Supplementary Information This file contains a list of sites and investigators; Supplementary Tables 1–4 and patient-reported outcomes

Reporting Summary

## Figures and Tables

**Extended Data Table 1:**
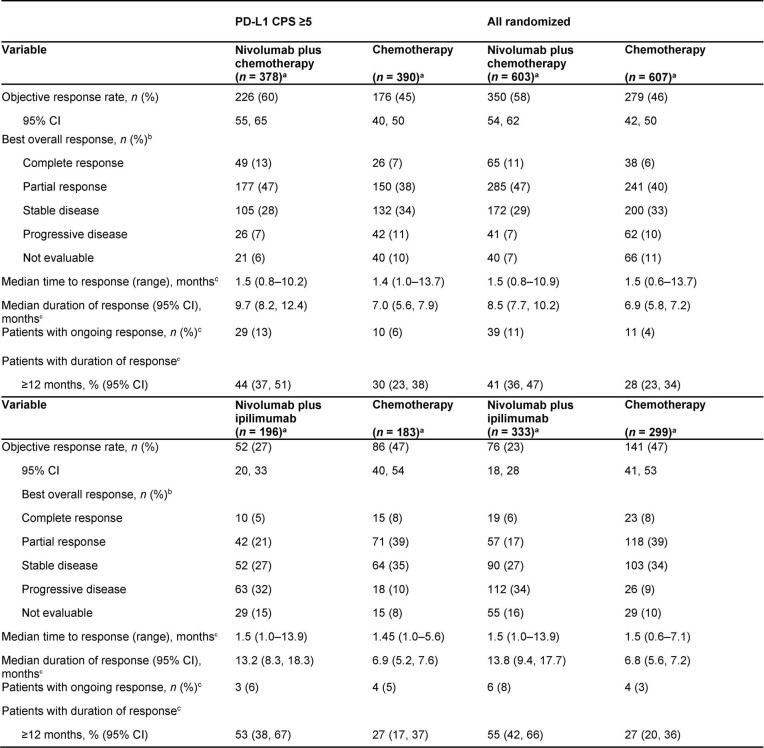
Response per BICR

^a^Randomized patients who had target lesion measurements at baseline, per BICR assessment; ^b^Percentages may not add up to 100% because of rounding; ^c^Evaluated in patients who had an objective response. BICR, blinded independent central review; CI, confidence interval; CPS, combined positive score; PD-L1, programmed death ligand 1.

## Data Availability

The Bristol Myers Squibb data sharing policy (https://www.bms.com/researchers-and-partners/independent-research/data-sharing-request-process.html) is compliant with ICMJE guidelines. Bristol Myers Squibb will honour legitimate requests for clinical trial data from qualified researchers. Data will be shared with external researchers whose proposed use of the data has been approved. Complete de-identified patient data sets will be eligible for sharing 2 years after completion of the CheckMate 649 study. Before data are released, the researcher(s) must sign a Data Sharing Agreement, after which the de-identified and anonymized datasets can be accessed within a secured portal.

## References

[1] Catenacci DVT (2017). Rilotumumab plus epirubicin, cisplatin, and capecitabine as first-line therapy in advanced MET-positive gastric or gastro-oesophageal junction cancer (RILOMET-1): a randomised, double-blind, placebo-controlled, phase 3 trial. Lancet Oncol..

[2] Fuchs CS (2019). Ramucirumab with cisplatin and fluoropyrimidine as first-line therapy in patients with metastatic gastric or junctional adenocarcinoma (RAINFALL): a double-blind, randomised, placebo-controlled, phase 3 trial. Lancet Oncol..

[3] Lordick F (2013). Capecitabine and cisplatin with or without cetuximab for patients with previously untreated advanced gastric cancer (EXPAND): a randomised, open-label phase 3 trial. Lancet Oncol..

[4] Shah MA (2017). Effect of fluorouracil, leucovorin, and oxaliplatin with or without onartuzumab in HER2-negative, MET-positive gastroesophageal adenocarcinoma: the METGastric randomized clinical trial. JAMA Oncol..

[5] Janjigian YY (2021). First-line nivolumab plus chemotherapy versus chemotherapy alone for advanced gastric, gastro-oesophageal junction, and oesophageal adenocarcinoma (CheckMate 649): a randomised, open-label, phase 3 trial. Lancet.

[6] *OPDIVO (Nivolumab) Injection for Intravenous Use. Prescribing Information* (Bristol Myers Squibb, 2021).

[7] Das R (2015). Combination therapy with anti-CTLA-4 and anti-PD-1 leads to distinct immunologic changes in vivo. J. Immunol..

[8] Brahmer JR (2010). Phase I study of single-agent anti-programmed death-1 (MDX-1106) in refractory solid tumors: safety, clinical activity, pharmacodynamics, and immunologic correlates. J. Clin. Oncol..

[9] Wang C (2014). In vitro characterization of the anti-PD-1 antibody nivolumab, BMS-936558, and in vivo toxicology in non-human primates. Cancer Immunol. Res..

[10] Pardoll DM (2012). The blockade of immune checkpoints in cancer immunotherapy. Nat. Rev. Cancer.

[11] Wei SC, Duffy CR, Allison JP (2018). Fundamental mechanisms of immune checkpoint blockade therapy. Cancer Discov..

[12] Janjigian YY (2018). CheckMate-032 study: efficacy and safety of nivolumab and nivolumab plus ipilimumab in patients with metastatic esophagogastric cancer. J. Clin. Oncol..

[13] Shitara K (2020). Efficacy and safety of pembrolizumab or pembrolizumab plus chemotherapy vs chemotherapy alone for patients with first-line, advanced gastric cancer: the KEYNOTE-062 phase 3 randomized clinical trial. JAMA Oncol..

[14] Lei M (2021). Analyses of PD-L1 and inflammatory gene expression association with efficacy of nivolumab ± ipilimumab in gastric cancer/gastroesophageal junction cancer. Clin. Cancer Res..

[15] Hagi T (2020). Multicentre biomarker cohort study on the efficacy of nivolumab treatment for gastric cancer. Br. J. Cancer.

[16] Fassan M (2020). PD-L1 expression in gastroesophageal dysplastic lesions. Virchows Arch..

[17] Xu J (2021). LBA53–Sintilimab plus chemotherapy (chemo) versus chemo as first-line treatment for advanced gastric or gastroesophageal junction (G/GEJ) adenocarcinoma (ORIENT-16): first results of a randomized, double-blind, phase III study. Ann. Oncol..

[18] Xu J, Jin Y, Liu Y, Zhou H, Wang Y (2019). ORIENT-16: sintilimab plus XELOX vs placebo plus XELOX as 1st line treatment for unresectable advanced gastric and GEJ adenocarcinoma. Cancer Res..

[19] Doki Y (2022). Nivolumab combination therapy in advanced esophageal squamous-cell carcinoma. N. Engl. J. Med.

[20] Motzer RJ (2018). Nivolumab plus ipilimumab versus sunitinib in advanced renal-cell carcinoma. N. Engl. J. Med..

[21] Baas P (2021). First-line nivolumab plus ipilimumab in unresectable malignant pleural mesothelioma (CheckMate 743): a multicentre, randomised, open-label, phase 3 trial. Lancet.

[22] Hellmann MD (2019). Nivolumab plus ipilimumab in advanced non-small-cell lung cancer. N. Engl. J. Med..

[23] Bang YJ (2018). Phase III, randomised trial of avelumab versus physician’s choice of chemotherapy as third-line treatment of patients with advanced gastric or gastro-oesophageal junction cancer: primary analysis of JAVELIN Gastric 300. Ann. Oncol..

[24] Shitara K (2018). Pembrolizumab versus paclitaxel for previously treated, advanced gastric or gastro-oesophageal junction cancer (KEYNOTE-061): a randomised, open-label, controlled, phase 3 trial. Lancet.

[25] Bass AJ (2014). Comprehensive molecular characterization of gastric adenocarcinoma. Nature.

[26] Cancer Genome Atlas Research Network (2017). Integrated genomic characterization of oesophageal carcinoma. Nature.

[27] Wolchok JD (2017). Overall survival with combined nivolumab and ipilimumab in advanced melanoma. N. Engl. J. Med..

[28] Angell HK (2019). PD-L1 and immune infiltrates are differentially expressed in distinct subgroups of gastric cancer. OncoImmunology.

[29] Le DT (2017). Mismatch repair deficiency predicts response of solid tumors to PD-1 blockade. Science.

[30] Zhou KI (2020). Spatial and temporal heterogeneity of PD-L1 expression and tumor mutational burden in gastroesophageal adenocarcinoma at baseline diagnosis and after chemotherapy. Clin. Cancer Res..

[31] Garland SN (2011). Prospective evaluation of the reliability, validity, and minimally important difference of the functional assessment of cancer therapy-gastric (FACT-Ga) quality-of-life instrument. Cancer.

[32] Kulangara K (2018). Investigation of PD-L1 expression and response to pembrolizumab (pembro) in gastric cancer (GC) and cervical cancer (CC) using combined positive score (CPS) and tumor proportion score (TPS). J Clin Oncol.

