# Supplementary material for: Nivolumab plus chemotherapy or ipilimumab in gastro-oesophageal cancer
Source: Nature. 2022 Mar 23;603(7903):942–8. doi: 10.1038/s41586-022-04508-4 (PMC8967713; doi:10.1038/s41586-022-04508-4)
Supplement: Supplementary file 1 — This file contains a list of sites and investigators; Supplementary Tables 1–4 and patient-reported outcomes [file 41586_2022_4508_MOESM1_ESM.pdf]

nature portfolio

https://doi.org/10.1038/s41586-022-04508-4

# Supplementary information

# Nivolumab plus chemotherapy or ipilimumab in gastro-oesophageal cancer

In the format provided by the authors and unedited

**SI Guide**

**Supplemental information to**

# Nivolumab plus chemotherapy or ipilimumab in gastroesophageal cancer

Shitara K, Ajani JA, Markus Moehler M, et al.

**Corresponding author:**

Yelena Y Janjigian, MD
Gastrointestinal Oncology Service, Department of Medicine
Memorial Sloan Kettering Cancer Center
New York, NY
Phone number: (+)1-646-888-4286
janjigiy@mskcc.org

**Table of Contents**

1. List of Sites and Investigators ........................................................................ 2

Principal investigators for each site who participated in this trial................................ 2

2. Supplemental Tables.................................................................................... 6

Supplemental Table 1 | Baseline demographics and clinical characteristics in all randomized patients.......................................................................................... 6

Supplemental Table 2 | Subsequent therapies ...................................................... 8

Supplemental Table 3 | Duration of treatment in all treated patients.......................... 9

Supplemental Table 4 | Summary of treatment-related adverse events and events with potential immunologic etiology in all treated patients......................................... 10

3. Patient-Reported Outcomes........................................................................... 12

## 1. LIST OF SITES AND INVESTIGATORS

### Principal investigators for each site who participated in this trial

| | |
|---|---|
| **Argentina** | Luciana Bellaquero |
| | Diego Kaen |
| | Ruben Kowalyszyn |
| | Guillermo Mendez |
| | Juan Manuel O’Connor |
| | Felipe Palazzo |
| | Eduardo Richardet |
| **Australia** | Sumitra Ananda |
| | Geoffrey Chong |
| | Kynan Feeney |
| | Andrew Hill |
| | Craig Kukard |
| | Adnan Nagrial |
| | Arvind Sahu |
| | Nimit Singhal |
| **Brazil** | Anelisa Araujo |
| | Maria Ignez Braghiroli |
| | Arinilda Campos Bragagnoli |
| | Sergio de Azevedo |
| | Fabio Franke |
| **Canada** | Jean-Sebastien Aucoin |
| | Felix Couture |
| | Elena Elimova |
| | Petr Kavan |
| | Frederic Lemay |
| | Mustapha Tehfe |
| | Mark Vincent |
| | Xiaofu Zhu |
| **Chile** | Olga Barajas |
| | Marcelo Garrido |
| | Eugenia Loredo Fort |
| | Pamela Salman |
| | Patricio Yanez |
| **China** | Yi Ba |
| | Chunmei Bai |
| | Li Bai |
| | Yuxian Bai |
| | Ying Cheng |
| | Weijian Guo |
| | Wenwei Hu |
| | Wei Li |
| | Xiaoyan Lin |

| | |
|---|---|
| | Tianshu Liu |
| | Jianwei Lu |
| | Hongming Pan |
| | Shukui Qin |
| | Lin Shen |
| | Yong Tang |
| | Jufeng Wang |
| | Nong Xu |
| | Ruihua Xu |
| | Nong Yang |
| | Jingdong Zhang |
| | Xiaochun Zhang |
| | Haijun Zhong |
| **Colombia** | Ricardo Bruges |
| | Carlos Narvaez |
| | Carlos Ortiz |
| **Czech Republic** | Zdenek Kral |
| | Jiri Tomasek |
| **France** | Romain Cohen |
| | Farid el Hajbi |
| | Pierre-Luc Etienne |
| | Ludovic Evesque |
| | Marie-Pierre Galais |
| | Francois Ghiringhelli |
| | Emmanuelle Samalin |
| | Yann Touchefeu |
| **Germany** | Heiko Becker |
| | Gunnar Folprecht |
| | Stefan Kasper-Virchow |
| | Verena Keitel |
| | Sylvie Lorenzen |
| | Markus Moehler |
| | Marianne Sinn |
| | Peter Thuss-Patience |
| | Thomas Zander |
| **Greece** | Gerasimos Aravantinos |
| | Haralabos Kalofonos |
| | Michalis Karamouzis |
| | George Pentheroudakis |
| **Hong Kong** | Ka On Lam |
| | Winnie Yeo |
| **Hungary** | Peter Arkosy |
| | Magdolna Dank |
| | Erika Hitre |
| **Israel** | Baruch Brenner |

| | |
|---|---|
| | Ravit Geva |
| | Ayala Hubert |
| | Maria Passhak |
| | Einat Shacham Shmueli |
| **Italy** | Michele Basso |
| | Alfredo Falcone |
| | Gabriele Luppi |
| | Evaristo Maiello |
| | Mario Mandala |
| | Vincenzo Montesarchio |
| **Japan** | Hiroki Hara |
| | Ken Kato |
| | Yoshito Komatsu |
| | Keiko Minashi |
| | Yukiya Narita |
| | Taroh Satoh |
| | Kohei Shitara |
| | Toshimi Takano |
| | Akihito Tsuji |
| | Kensei Yamaguchi |
| **Mexico** | Adriana Dominguez Andrade |
| | Angel Gomez Villanueva |
| | Alejandro Juarez Ramiro |
| **Peru** | Ashley Alarcon |
| | Paola Montenegro |
| | Renzo Salas |
| | Hermes Tejada |
| **Poland** | Iwona Drab-Mazur |
| | Tomasz Skoczylas |
| | Lucjan Wyrwicz |
| **Portugal** | Antonio Quintela |
| | Nuno Sousa |
| **Romania** | Adina Croitoru |
| | Filip Dumitru |
| | Doina Elena Ganea |
| | Anca Mihailov |
| | Michael Schenker |
| **Russia** | Larisa Bolotina |
| | Oleg Gladkov |
| | Nina Karaseva |
| | Elena Poddubskaya |
| | Sergey Tjulandin |
| **Singapore** | Chau Hsien Matthew Ng |
| | Wei-Peng Yong |
| **South Korea** | Hyun Cheol Chung |

| | |
|---|---|
| | Yoon Koo Kang |
| **Spain** | Maria Alsina |
| | Cristina Buges |
| | Andres Cervantes |
| | Ignacio Delgado |
| | Jose Federico Gonzalez |
| | Roberto Antonio Pazo Cid |
| **Taiwan** | Yee Chao |
| | Jen-Shi Chen |
| **Turkey** | Irfan Cicin |
| | Hasan Coskun |
| | Ali Kaplan |
| | Mehmet Ali Nahit Sendur |
| | Dogan Uncu |
| **UK** | Ian Chau |
| | Tim Iveson |
| | Srinivasan Madhusudan |
| | Wasat Mansoor |
| **USA** | Jaffer Ajani |
| | Ari Baron |
| | Johanna Bendell |
| | David Chang |
| | James Cleary |
| | Allen Cohn |
| | Jay Courtright |
| | Sarah Davis |
| | Joseph Fiorillo |
| | Sunil Gandhi |
| | Henrik Bo Illum |
| | Syma Iqbal |
| | Yelena Janjigian |
| | Blair Jobe |
| | Alok Khorana |
| | Kristen Marrone |
| | Steven McCune |
| | Andrew Scott Paulson |
| | James Reeves |
| | Joel Saltzman |
| | Usman Shah |
| | Richard Siegel |
| | Antonio Ucar |
| | Victoria Villaflor |
| | Benjamin Weinberg |

## 2. SUPPLEMENTAL TABLES

### Supplemental Table 1 | Baseline demographics and clinical characteristics in all randomized patients

| | **Nivolumab plus chemotherapy (*n* = 789)[a]** | **Chemotherapy (*n* = 792)[a]** | **Nivolumab plus ipilimumab (*n* = 409)[b]** | **Chemotherapy (*n* = 404)[b]** |
|---|---|---|---|---|
| Age (years), median (range) | 62 (18–88) | 61 (21–90) | 62 (22–84) | 61 (23–90) |
| <65 | 473 (60) | 488 (62) | 258 (63) | 251 (62) |
| ≥65 | 316 (40) | 304 (38) | 151 (37) | 153 (38) |
| Sex, *n* (%) | | | | |
| Male | 540 (68) | 560 (71) | 278 (68) | 280 (69) |
| Female | 249 (32) | 232 (29) | 131 (32) | 124 (31) |
| Race, *n* (%) | | | | |
| Asian | 186 (24) | 189 (24) | 124 (30) | 121 (30) |
| White | 556 (70) | 541 (68) | 264 (65) | 263 (65) |
| Other[c] | 47 (6) | 62 (8) | 21 (5) | 20 (5) |
| Region, *n* (%) | | | | |
| Asia | 178 (23) | 178 (22) | 118 (29) | 117 (29) |
| USA and Canada | 131 (17) | 132 (17) | 75 (18) | 69 (17) |
| Rest of the world | 480 (61) | 482 (61) | 216 (53) | 218 (54) |
| ECOG PS,[d] *n* (%) | | | | |
| 0 | 326 (41) | 336 (42) | 180 (44) | 185 (46) |
| 1 | 462 (59) | 452 (57) | 228 (56) | 219 (54) |
| Primary tumor location at initial diagnosis, *n* (%) | | | | |
| Gastric cancer | 554 (70) | 556 (70) | 282 (69) | 282 (70) |
| GEJ cancer | 132 (17) | 128 (16) | 82 (20) | 73 (18) |
| Esophageal adenocarcinoma | 103 (13) | 108 (14) | 45 (11) | 49 (12) |
| Tumor cell PD-L1 expression, *n* (%) | | | | |
| <1%[e] | 663 (84) | 664 (84)[f] | 341 (83) | 337 (83) |
| ≥1% | 126 (16) | 127 (16) | 68 (17) | 67 (17) |
| Previous surgery, *n* (%) | | | | |
| Yes | 160 (20) | 176 (22) | 86 (21) | 95 (24) |
| No | 629 (80) | 616 (78) | 323 (79) | 309 (76) |
| Organs with metastases, *n* (%) | | | | |
| 1 | 164 (21) | 183 (23) | 83 (20) | 100 (25) |
| ≥2 | 602 (76) | 583 (74) | 326 (80) | 304 (75) |
| Liver metastases,[g] *n* (%) | | | | |
| Yes | 301 (38) | 314 (40) | 149 (36) | 160 (40) |
| No | 465 (59) | 452 (57) | 255 (62) | 228 (56) |
| Signet ring cell carcinoma,[h] *n* (%) | | | | |
| Yes | 145 (18) | 136 (17) | 71 (17) | 83 (21) |
| No | 644 (82) | 656 (83) | 338 (83) | 321 (79) |
| Lauren's classification, *n* (%) | | | | |
| Intestinal type | 272 (34) | 267 (34) | 134 (33) | 119 (29) |
| Diffuse type | 254 (32) | 273 (34) | 143 (35) | 160 (40) |
| Mixed | 58 (7) | 48 (6) | 20 (5) | 28 (7) |
| Unknown | 205 (26) | 204 (26) | 112 (27) | 97 (24) |
| Microsatellite instability status,[i] *n* (%) | | | | |
| MSS | 695 (88) | 682 (86) | 355 (87) | 344 (85) |
| MSI-H | 23 (3) | 21 (3) | 11 (3) | 10 (2) |
| Chemotherapy regimen,[j] *n* (%) | | | | |
| FOLFOX | 422/782 (54) | 406/767 (53) | NA | 181/389 (47) |
| CapeOX | 360/782 (46) | 361/767 (47) | NA | 208/389 (53) |
| Albumin, *n* (%) | | | | |
| <LLN | 179 (23) | 178 (22) | 89 (22) | 74 (18) |

| | | | | |
|---|---|---|---|---|
| ≥LLN | 578 (73) | 581 (73) | 311 (76) | 316 (78) |
| Not reported | 32 (4) | 33 (4) | 9 (2) | 14 (3) |
| Previous adjuvant or neoadjuvant therapy, *n* (%) | | | | |
| Adjuvant | 63 (8) | 56 (7) | 22 (5) | 31 (8) |
| Neoadjuvant | 48 (6) | 61 (8) | 27 (7) | 33 (8) |
| Metastatic disease | 1 (<1) | 0 | 0 | 0 |
| Disease stage, *n* (%) | | | | |
| Metastatic | 757 (96) | 756 (95) | 391 (96) | 386 (96) |
| Locally advanced | 27 (3) | 34 (4) | 14 (3) | 18 (4) |
| Locally recurrent | 5 (1) | 2 (<1) | 4 (1) | 0 |
| PD-L1 CPS expression, *n* (%) | | | | |
| <1 | 140 (18) | 125 (16) | 80 (20) | 77 (19) |
| ≥1 | 641 (81) | 656 (83) | 322 (79) | 319 (79) |
| <5 | 308 (39) | 299 (38) | 168 (41) | 157 (39) |
| ≥5 | 473 (60) | 482 (61) | 234 (57) | 239 (59) |
| <10 | 406 (51) | 389 (49) | 221 (55) | 198 (50) |
| ≥10 | 375 (48) | 392 (49) | 181 (45) | 198 (50) |
| Not evaluable/ indeterminate/missing | 8 (1) | 11 (1) | 7 (2) | 8 (2) |
| Site of metastases, *n* (%) | | | | |
| Liver | 301 (38) | 314 (40) | 149 (36) | 160 (40) |
| Peritoneum | 188 (24) | 188 (24) | 95 (23) | 98 (24) |
| CNS | 1 (<1) | 0 | 0 | 0 |

[a]Concurrently randomized in nivolumab plus chemotherapy versus chemotherapy; [b]Concurrently randomized in nivolumab plus ipilimumab versus chemotherapy; [c]Included American Indian or Alaska Native (nivolumab plus chemotherapy versus chemotherapy, *n* = 26; nivolumab plus ipilimumab versus chemotherapy, *n* = 3), Black or African American (nivolumab plus chemotherapy versus chemotherapy, *n* = 18; nivolumab plus ipilimumab versus chemotherapy, *n* = 9) and not reported (nivolumab plus chemotherapy versus chemotherapy, *n* = 1); [d]Based on case report form. ECOG PS of 2 was reported in four patients in the nivolumab plus chemotherapy versus chemotherapy group and one patient in the nivolumab-plus-ipilimumab versus chemotherapy group. ECOG PS was not reported for one patient in the nivolumab plus chemotherapy versus chemotherapy group. All randomly assigned patients had ECOG PS of 0 or 1 based on interactive response technology; [e]Includes indeterminate tumor cell PD-L1 expression; [f]One patient had missing PD-L1 expression at baseline; [g]Liver metastases not reported for 49 patients in the nivolumab plus chemotherapy versus chemotherapy group and 21 patients in the nivolumab plus ipilimumab versus chemotherapy group; [h]Per WHO histological classification; [i]MSI status was not reported or invalid for 160 patients in the nivolumab plus chemotherapy versus chemotherapy group and 93 patients in the nivolumab plus ipilimumab versus chemotherapy group; [j]Patients who received at least one dose of the assigned treatment. CapeOX, capecitabine plus oxaliplatin; CNS, central nervous system; CPS, combined positive score; ECOG PS, Eastern Cooperative Oncology Group performance status; FOLFOX, 5-fluorouracil plus leucovorin plus oxaliplatin; GEJ, gastroesophageal junction; LLN, lower limit of normal; MSI, microsatellite instability; MSI-H, microsatellite instability-high; MSS, microsatellite stable; NA, not applicable; PD-L1, programmed death ligand 1.

**Supplemental Table 2 | Subsequent therapies**

| | Nivolumab plus chemotherapy (*n* = 789) | Chemotherapy (*n* = 792) | Nivolumab plus ipilimumab (*n* = 409) | Chemotherapy (*n* = 404) |
|---|---|---|---|---|
| Any subsequent therapy[a] | 325 (41) | 346 (44) | 196 (48) | 187 (46) |
| Subsequent radiotherapy | 43 (5) | 48 (6) | 26 (6) | 25 (6) |
| Subsequent surgery | 20 (3) | 26 (3) | 13 (3) | 11 (3) |
| Subsequent systemic anti-cancer therapy | 290 (37) | 329 (42) | 186 (45) | 179 (44) |
| Most frequent systemic anti-cancer therapies | | | | |
| Chemotherapy | 281 (36) | 306 (39) | 179 (44) | 167 (41) |
| Taxanes | | | | |
| Paclitaxel | 165 (21) | 185 (23) | 58 (14) | 105 (26) |
| Docetaxel | 20 (3) | 23 (3) | 15 (4) | 18 (4) |
| Fluoropyrimidine-based chemotherapy | | | | |
| Fluorouracil | 78 (10) | 108 (14) | 119 (29) | 57 (14) |
| Capecitabine | 28 (4) | 25 (3) | 28 (7) | 14 (3) |
| Fluoropyrimidine | 0 | 2[b] (<1) | – | – |
| Platinum-based chemotherapy | | | | |
| Oxaliplatin | 32 (4) | 29 (6) | 146 (36) | 29 (7) |
| Carboplatin | 9 (1) | 10 (1) | 6 (1) | 7 (2) |
| Cisplatin | 15 (2) | 17 (2) | 10 (2) | 14 (3) |
| Targeted therapy | | | | |
| Ramucirumab | 101 (13) | 96 (12) | 29 (7) | 65 (16) |
| Apatinib | 13 (2) | 21 (3) | 7 (2) | 16 (4) |
| Immunotherapy | 17 (2) | 73 (9) | 11 (3) | 47 (12) |
| Nivolumab | 10 (1) | 35 (4) | 6 (1) | 28 (7) |
| Pembrolizumab | 3 (<1) | 29 (4) | 4 (1) | 18 (4) |
| Atezolizumab | 0 | 4 (<1) | – | – |
| Toripalimab | 1 (<1) | 3 (<1) | 0 | 1 (<1) |
| Ipilimumab | 1 (<1) | 2 (<1) | 0 | 1 (<1) |
| Other | 3 (<1) | 3 (<1) | 2 (<1) | 1 (<1) |

Data are presented as *n* (%). [a]Patients could have received more than one type of therapy; [b]Includes one patient who received floxuridine.

**Supplemental Table 3 | Duration of treatment in all treated patients**

| Treatment | Median duration of treatment, months (range) | Dose reduction or omission, *n* (%) |
|---|---|---|
| Nivolumab plus chemotherapy (*n* = 782) | 6.8 (0.1–45.0) | – |
| Nivolumab plus CapeOX (*n* = 360) | | |
| Nivolumab[a] (mg) | 5.5 (0.0–24.1) | – |
| Oxaliplatin[b] (mg/m$^2$) | 4.0 (0.0–23.2) | 167 (46) |
| Capecitabine[c] (mg/m$^2$) | 5.7 (0.1–45.0) | 53 (15) |
| Nivolumab plus FOLFOX (*n* = 422) | | – |
| Nivolumab[a] (mg) | 6.7 (0.0–26.0) | – |
| Oxaliplatin[b] (mg/m$^2$) | 4.6 (0.0–23.5) | 181 (43) |
| 5-Fluorouracil bolus[b] (mg/m$^2$) | 5.3 (0.0–35.2) | 140 (33) |
| 5-Fluorouracil continuous[b] (mg/m$^2$) | 5.9 (0.1–35.2) | 181 (43) |
| Chemotherapy (*n* = 767) | 4.9 (0.0–44.2) | – |
| CapeOX (*n* = 361) | | |
| Oxaliplatin[b] (mg/m$^2$) | 3.7 (0.0–34.4) | 145 (40) |
| Capecitabine[c] (mg/m$^2$) | 4.8 (0.0–44.2) | 56 (16) |
| FOLFOX (*n* = 406) | | |
| Oxaliplatin[b] (mg/m$^2$) | 4.2 (0.0–35.4) | 181 (45) |
| 5-Fluorouracil bolus[b] (mg/m$^2$) | 4.4 (0.0–42.9) | 148 (37) |
| 5-Fluorouracil continuous[b] (mg/m$^2$) | 4.9 (0.1–42.9) | 152 (37) |
| Nivolumab plus ipilimumab (*n* = 403) | 1.9 (0.0–24.1) | – |
| Nivolumab[a] (mg) | 2.5 (0.7–24.5) | – |
| Ipilimumab[a] (mg) | 1.4 (0.0–5.0) | – |
| Chemotherapy | 4.9 (0.1–45.5) | – |
| CapeOX (*n* = 208) | 4.9 (0.1–44.2) | – |
| Oxaliplatin[b] (mg/m$^2$) | 3.8 (0.0–34.4) | 91 (44) |
| Capecitabine[c] (mg/m$^2$) | 4.9 (0.1–44.2) | 41 (20) |
| FOLFOX (*n* = 181) | 4.7 (0.1–45.5) | – |
| Oxaliplatin[b] (mg/m$^2$) | 4.1 (0.0–35.4) | 83 (46) |
| 5-Fluorouracil bolus[b] (mg/m$^2$) | 4.3 (0.0–45.4) | 66 (37) |
| 5-Fluorouracil continuous[b] (mg/m$^2$) | 4.7 (0.1–45.5) | 69 (38) |

[a]Dose reductions were not allowed for nivolumab or ipilimumab; [b]Includes patients with at least one dose reduction; [c]Includes patients with at least one omitted dose. FOLFOX, 5-fluorouracil plus leucovorin plus oxaliplatin; CapeOX, capecitabine plus oxaliplatin.

**Supplemental Table 4 | Summary of treatment-related adverse events and events with potential immunologic etiology in all treated patients**

| | Nivolumab plus chemotherapy ($n$ = 782)[a,b] | | Chemotherapy ($n$ = 767)[a,b] | | Nivolumab plus ipilimumab ($n$ = 403)[a,c] | | Chemotherapy ($n$ = 389)[a,c] | |
|---|---|---|---|---|---|---|---|---|
| | Any grade | Grade 3–4 | Any grade | Grade 3–4 | Any grade | Grade 3–4 | Any grade | Grade 3–4 |
| **Events in 10% or more of treated patients in either group** | | | | | | | | |
| Nausea | 328 (42) | 21 (3) | 300 (39) | 19 (2) | 38 (9) | 6 (1) | 179 (46) | 16 (4) |
| Diarrhea | 258 (33) | 35 (4) | 207 (27) | 24 (3) | 72 (18) | 11 (3) | 112 (29) | 15 (4) |
| Neuropathy peripheral | 225 (29) | 33 (4) | 193 (25) | 22 (3) | 3 (<1) | 1 (<1) | 93 (24) | 9 (2) |
| Anemia | 205 (26) | 47 (6) | 173 (23) | 20 (3) | 30 (7) | 5 (1) | 85 (22) | 9 (2) |
| Fatigue | 206 (26) | 30 (4) | 174 (23) | 18 (2) | 64 (16) | 11 (3) | 85 (22) | 8 (2) |
| Vomiting | 198 (25) | 17 (2) | 170 (22) | 24 (3) | 22 (5) | 4 (1) | 86 (22) | 11 (3) |
| Neutropenia | 193 (25) | 121 (15) | 185 (24) | 96 (13) | 6 (1) | 1 (<1) | 93 (24) | 44 (11) |
| Decreased appetite | 158 (20) | 14 (2) | 139 (18) | 13 (2) | 41 (10) | 5 (1) | 89 (23) | 6 (2) |
| Neutrophil count decreased | 159 (20) | 84 (11) | 118 (15) | 67 (9) | 5 (1) | 1 (<1) | 69 (18) | 40 (10) |
| Thrombocytopenia | 159 (20) | 21 (3) | 149 (19) | 14 (2) | 6 (1) | 1 (<1) | 70 (18) | 7 (2) |
| Platelet count decreased | 158 (20) | 20 (3) | 115 (15) | 19 (2) | 6 (1) | 2 (<1) | 59 (15) | 11 (3) |
| Peripheral sensory neuropathy | 137 (18) | 16 (2) | 119 (16) | 14 (2) | 1 (<1) | 0 | 54 (14) | 6 (2) |
| Aspartate aminotransferase increased | 123 (16) | 13 (2) | 70 (9) | 5 (<1) | 53 (13) | 16 (4) | 36 (9) | 2 (<1) |
| White blood cell count decreased | 114 (15) | 23 (3) | 77 (10) | 13 (2) | 4 (1) | 0 | 52 (13) | 9 (2) |
| Alanine aminotransferase increased | 90 (12) | 7 (<1) | 51 (7) | 5 (<1) | 52 (13) | 16 (4) | 27 (7) | 1 (<1) |
| Palmar-plantar erythrodysaesthesia syndrome | 97 (12) | 12 (2) | 83 (11) | 8 (1) | 0 | 0 | 47 (12) | 2 (<1) |
| Lipase increased | 89 (11) | 45 (6) | 34 (4) | 16 (2) | 45 (11) | 28 (7) | 21 (5) | 11 (3) |
| Rash | 77 (10) | 7 (<1) | 12 (2) | 0 | 59 (15) | 10 (2) | 8 (2) | 0 |
| Asthenia | 76 (10) | 7 (<1) | 83 (11) | 10 (1) | 28 (7) | 2 (<1) | 34 (9) | 5 (1) |
| Hypothyroidism | 71 (9) | 0 | 2 (<1) | 0 | 48 (12) | 0 | 2 (<1) | 0 |
| Amylase increased | 71 (9) | 21 (3) | 22 (3) | 2 (<1) | 42 (10) | 18 (4) | 15 (4) | 3 (<1) |
| Pyrexia | 64 (8) | 4 (<1) | 22 (3) | 1 (<1) | 42 (10) | 1 (<1) | 12 (3) | 0 |
| **Events with potential immunologic etiology in all treated patients[a,d]** | | | | | | | | |
| Endocrine | 109 (14) | 6 (<1) | 3 (<1) | 0 | 89 (22) | 15 (4) | 2 (<1) | 0 |
| Gastrointestinal | 266 (34) | 43 (5) | 208 (27) | 25 (3) | 84 (21) | 26 (6) | 113 (29) | 16 (4) |
| Hepatic | 207 (26) | 31 (4) | 138 (18) | 17 (2) | 104 (26) | 47 (12) | 74 (19) | 9 (2) |
| Pulmonary | 41 (5) | 14 (2) | 4 (<1) | 1 (<1) | 15 (4) | 4 (1) | 1 (<1) | 0 |
| Renal | 29 (4) | 7 (<1) | 9 (1) | 2 (<1) | 17 (4) | 6 (1) | 6 (2) | 1 (<1) |
| Skin | 218 (28) | 27 (3) | 108 (14) | 8 (1) | 146 (36) | 17 (4) | 55 (14) | 2 (<1) |

Data are presented as $n$ (%). [a]Patients who received at least one dose of the assigned treatment. Includes events reported between first dose and 30 days after last dose of trial therapy. Treatment-relatedness in the nivolumab plus chemotherapy group refers to nivolumab, at least one chemotherapy

component or both and in the nivolumab plus ipilimumab group to nivolumab, ipilimumab or both. National Cancer Institute Common Terminology Criteria for Adverse Events, version 4.0, and Medical Dictionary for Regulatory Activities, version 23.0; [b]Concurrently randomized to nivolumab plus chemotherapy versus chemotherapy; [c]Concurrently randomized to nivolumab plus ipilimumab versus chemotherapy; [d]Treatment-related select adverse events by organ category that have potential immunologic etiology and require frequent monitoring/intervention.

## 3. PATIENT-REPORTED OUTCOMES

Among patients with PD-L1 CPS ≥5 and all-randomized patients who were eligible for PRO assessments, the proportion completing the Functional Assessment of Cancer Therapy-Gastric (FACT-Ga) questionnaire in both treatment arms was 90% or more at baseline and 80% or more at most subsequent assessments for which at least 10 patients were eligible (until week 133). Mean total scores at baseline were similar between the nivolumab-plus-chemotherapy and chemotherapy alone groups in PD-L1 CPS ≥5 and all-randomized patients.[1]

### Reference

1. Janjigian, Y. Y. et al. First-line nivolumab plus chemotherapy versus chemotherapy alone for advanced gastric, gastro-oesophageal junction, and oesophageal adenocarcinoma (CheckMate 649): a randomised, open-label, phase 3 trial. *Lancet* **398**, 27-40 (2021).
