# Supplementary material for: Nivolumab plus chemotherapy or ipilimumab in gastro-oesophageal cancer
Source: Nature. 2022 Mar 23;603(7903):942–8. doi: 10.1038/s41586-022-04508-4 (PMC8967713; doi:10.1038/s41586-022-04508-4)
Supplement: Supplementary file 2 — Reporting Summary [file 41586_2022_4508_MOESM2_ESM.pdf]

nature portfolio

Corresponding author(s): Yelena Y Janjigian

Last updated by author(s): Jan 20, 2022

Reporting Summary

Nature Portfolio wishes to improve the reproducibility of the work that we publish. This form provides structure for consistency and transparency in reporting. For further information on Nature Portfolio policies, see our Editorial Policies and the Editorial Policy Checklist.

Statistics

For all statistical analyses, confirm that the following items are present in the figure legend, table legend, main text, or Methods section.

n/a	Confirmed	
☐	☒	The exact sample size (n) for each experimental group/condition, given as a discrete number and unit of measurement
☒	☐	A statement on whether measurements were taken from distinct samples or whether the same sample was measured repeatedly
☐	☒	The statistical test(s) used AND whether they are one- or two-sided<br>*Only common tests should be described solely by name; describe more complex techniques in the Methods section.*
☒	☐	A description of all covariates tested
☐	☒	A description of any assumptions or corrections, such as tests of normality and adjustment for multiple comparisons
☐	☒	A full description of the statistical parameters including central tendency (e.g. means) or other basic estimates (e.g. regression coefficient) AND variation (e.g. standard deviation) or associated estimates of uncertainty (e.g. confidence intervals)
☐	☒	For null hypothesis testing, the test statistic (e.g. F, t, r) with confidence intervals, effect sizes, degrees of freedom and P value noted<br>*Give P values as exact values whenever suitable.*
☒	☐	For Bayesian analysis, information on the choice of priors and Markov chain Monte Carlo settings
☐	☒	For hierarchical and complex designs, identification of the appropriate level for tests and full reporting of outcomes
☒	☐	Estimates of effect sizes (e.g. Cohen's d, Pearson's r), indicating how they were calculated

Our web collection on statistics for biologists contains articles on many of the points above.

Software and code

Policy information about availability of computer code

Data collection	Sample size calculations of the primary endpoints were based on simulations in East software, version 6.4.1. Eligible patients were randomized using interactive web response technology (block sizes of six) and assigned a unique patient number. All observations and other data pertinent to the investigation on each individual treated or entered as a control in the investigation were recorded on (electronic) case report forms. De-identified patient data was analyzed by the sponsor. Clinical data was entered into the Oracle Clinical database (TAO, Trial Access Online) through completion of the eCRF by clinical trial sites and through external data loading in the case of vendor data (eg, PD L1 CPS data). Separately, BICR data from BioClinica was loaded directly into the statistical computing environment (UNIX, Linux) for analysis purposes.
Data analysis	Overall tumor burden and measurable disease were assessed using Radiological Evaluation Criteria in Solid Tumors version 1.1 (RECIST CRITERIA 1.1). Statistical analyses were performed using SAS software, version 9.4 (SAS Institute, Cary, NC).

For manuscripts utilizing custom algorithms or software that are central to the research but not yet described in published literature, software must be made available to editors and reviewers. We strongly encourage code deposition in a community repository (e.g. GitHub). See the Nature Portfolio guidelines for submitting code & software for further information.

Data

Policy information about availability of data

All manuscripts must include a data availability statement. This statement should provide the following information, where applicable:

- Accession codes, unique identifiers, or web links for publicly available datasets
- A description of any restrictions on data availability
- For clinical datasets or third party data, please ensure that the statement adheres to our policy

The Bristol Myers Squibb data sharing policy can be found online and is compliant with ICMJE guidelines. Bristol Myers Squibb will honor legitimate requests for clinical trial data from qualified researchers. Data will be shared with external researchers whose proposed use of the data has been approved. Complete de-identified patient data sets will be eligible for sharing 2 years after completion of the CheckMate 649 study. Before data are released, the researcher(s) must sign a Data Sharing Agreement, after which the de-identified and anonymised datasets can be accessed within a secured portal. For the Bristol Myers Squibb policy on data sharing, see
https://www.bms.com/researchers-and-partners/independent-research/data-sharing-request-process.html.

Field-specific reporting

Please select the one below that is the best fit for your research. If you are not sure, read the appropriate sections before making your selection.

☒ Life sciences ☐ Behavioural & social sciences ☐ Ecological, evolutionary & environmental sciences

For a reference copy of the document with all sections, see nature.com/documents/nr-reporting-summary-flat.pdf

Life sciences study design

All studies must disclose on these points even when the disclosure is negative.

Sample size	Sample size calculations of the primary endpoints were based on simulations in East software, version 6.4.1. The statistical power estimation for the comparison of primary endpoints for the nivolumab plus chemotherapy versus the chemotherapy groups has been described previously in Janjigian, Y. Y. et al. Lancet 398, 27-40 (2021). The prevalence of patients with PD-L1 CPS ≥5 was assumed to be 35% of all randomized patients, based on limited available data, with 285 patients estimated in the nivolumab plus ipilimumab versus chemotherapy analysis. Based on new information from the CheckMate 649 trial, this PD-L1 CPS ≥5 prevalence was revised to 60% of all randomized patients, with 489 patients estimated in the nivolumab plus ipilimumab versus chemotherapy analysis. For OS, the HR was modeled as a four-piece HR with an average of 0.7. With a minimum follow-up of 36 months, it was expected that the 411 events would provide 93% power.
Data exclusions	No data were excluded from the analyses.
Replication	Not applicable as this paper presents results of a clinical trial.
Randomization	Randomization was done using interactive web response technology (block sizes of six) and stratified according to tumor cell PD-L1 status (≥1% vs <1% or indeterminate), region (Asia vs USA and Canada vs rest of world), Eastern Cooperative Oncology Group performance status (0 vs 1), and type of chemotherapy (CapeOX vs FOLFOX).
Blinding	Not applicable - open label study

Reporting for specific materials, systems and methods

We require information from authors about some types of materials, experimental systems and methods used in many studies. Here, indicate whether each material, system or method listed is relevant to your study. If you are not sure if a list item applies to your research, read the appropriate section before selecting a response.

Materials & experimental systems

n/a	Involved in the study	
☒	☐	Antibodies
☒	☐	Eukaryotic cell lines
☒	☐	Palaeontology and archaeology
☒	☐	Animals and other organisms
☐	☒	Human research participants
☐	☒	Clinical data
☒	☐	Dual use research of concern

Methods

n/a	Involved in the study	
☒	☐	ChIP-seq
☒	☐	Flow cytometry
☒	☐	MRI-based neuroimaging

Human research participants

Policy information about studies involving human research participants

Population characteristics	Males and females ≥ 18 years of age with unresectable advanced or metastatic gastric, GEJ or esophageal adenocarcinoma were enrolled, regardless of PD-L1 expression. Patients with known HER2-positive status were excluded, and prior systemic therapy for metastatic disease was not allowed. Randomization was stratified according to tumor cell PD-L1 status (≥1% vs <1% or indeterminate), region (Asia vs USA and Canada vs rest of world), Eastern Cooperative Oncology Group performance status (0 vs 1), and type of chemotherapy (CapeOX vs FOLFOX). Please see the manuscript methods section and protocol for additional details.
Recruitment	CheckMate 649 (NCT02872116) was conducted at 175 hospitals and cancer centers in 29 countries across Asia, Australia, Europe, North America, and South America. Overall, 3,185 patients were enrolled, and 2,031 were randomized; of these, 1,581 patients were concurrently randomized to nivolumab plus chemotherapy (789 patients) or chemotherapy (792 patients) (from April 2017 to May 2019), and 813 were concurrently randomized to nivolumab plus ipilimumab (409 patients) or chemotherapy (404 patients) (from October 2016 to June 2018). Once informed consent obtained from a prospective patient, they were enrolled using the Interactive Response Technology (IRT) system. After all eligibility criteria were met, the decision of chemotherapy regimen made, and PD-L1 test results from central lab was available in the IRT (both the site and the BMS study team remain blinded to the result), the site could conduct the IRT for randomization. The randomization was a block randomization stratified by region thus, it is unlikely that patient or investigator could bias treatment assignment. The investigator and patient were also blinded to PD-L1 CPS results.
Ethics oversight	The trial was conducted according to Good Clinical Practice guidelines developed by the International Council for Harmonisation and in compliance with the trial protocol. The trial protocol was approved by the institutional review boards or independent ethics committees at each site (NCT02872116). All patients provided written informed consent prior to trial participation per Declaration of Helsinki principles.

Note that full information on the approval of the study protocol must also be provided in the manuscript.

Clinical data

Policy information about clinical studies

All manuscripts should comply with the ICMJE guidelines for publication of clinical research and a completed CONSORT checklist must be included with all submissions.

Clinical trial registration	NCT02872116
Study protocol	The study protocol and statistical analysis plan has been submitted as a supplemental file along with the manuscript. Proprietary information has been redacted in these documents as allowed by journal guidelines
Data collection	CheckMate 649 was conducted at 175 hospitals and cancer centers in 29 countries across Asia, Australia, Europe, North America, and South America. Patients were enrolled and randomized across the 3 treatment groups; randomization to nivolumab plus chemotherapy or chemotherapy occurred from April 2017 to May 2019 and to nivolumab plus ipilimumab or chemotherapy from October 2016 to June 2018. Data cutoff was May 27, 2021.
Outcomes	The dual primary endpoints were OS (time from randomization to death) and PFS (time from randomization to the date of the first documented tumor progression (by blinded independent central review (BICR) per RECIST, version 1.1) or death) in the nivolumab plus chemotherapy versus chemotherapy groups in patients with PD-L1 CPS ≥5. Secondary endpoints that were hierarchically tested if the primary endpoints were met were OS in patients with PD-L1 CPS ≥1 and in all randomized patients in the nivolumab plus chemotherapy versus chemotherapy group and OS and TTSD in patients with PD-L1 CPS ≥5 and in all randomized patients in the nivolumab plus ipilimumab versus chemotherapy group. Please see the manuscript methods section and protocol for additional details.
